# Engraftment Outcome of CRISPR/Cas9-Edited Hematopoietic Stem Cells for Genetic Diseases: A Systematic Review and Meta-Analysis of Preclinical Evidence

**DOI:** 10.14740/jh2190

**Published:** 2026-04-06

**Authors:** Sudhanshu Yadav, Bandana Chakravarti, Baby Anjum, Shubhanshu Yadav, Prashant Kumar Singh, Ashok Kumar

**Affiliations:** a Stem Cell Research Centre, Department of Hematology, Sanjay Gandhi Postgraduate Institute of Medical Sciences, Lucknow, Uttar Pradesh 226014, India; b Stem Cell/Cell Culture Lab Unit, Centre for Advance Research, King George’s Medical University, Lucknow 226003, India; c Department of Neurology, Sanjay Gandhi Postgraduate Institute of Medical Sciences, Lucknow 226014, India.; d IQVIA, Marathahalli, Bengaluru 560103, India; e Department of Biochemistry, University of Lucknow, Lucknow 226007, India; f Department of Biochemistry and Biophysics, Rochester University Medical Centre, Rochester, NY 14625, USA

**Keywords:** Hematopoietic stem cell, Gene editing, Transplantation, Mice model, Engraftment efficiency, Peripheral blood, Bone marrow, Spleen

## Abstract

**Background:**

CRISPR-Cas9 (clustered regularly interspaced short palindromic repeats and CRISPR-associated protein 9)-based gene editing represents a promising frontier for treating monogenic hematologic disorders. Several preclinical studies have demonstrated the transplantation efficiency of CRISPR-Cas9-mediated gene editing in hematopoietic stem and progenitor cells (HSPCs) using various animal models. Nonetheless, these studies have employed diverse gene-editing strategies, utilizing HSPCs from different origins and transplanting them into distinct mouse strains. The present study aimed to determine the optimum conditions for efficient engraftment of genetically modified HSPCs across various organs, thereby facilitating the translation of preclinical research into clinical applications.

**Methods:**

We conducted a comprehensive literature search using PubMed Medline, Web of Science, and Google Scholar for relevant articles published from 2014 to 2025 that evaluated the engraftment potential of CRISPR-Cas9 HSPCs in genetic disease models. A total of 39 studies met the inclusion criteria and were included in a meta-analysis using Jamovi software.

**Results:**

The study revealed a significantly reduced engraftment of gene-edited cells in the bone marrow, spleen, and peripheral blood in the pooled analysis. Subgroup analyses revealed that knockout cells exhibited diminished engraftment, whereas knock-in cells demonstrated engraftment levels comparable to those of their non-edited counterparts. No evidence of publication bias or substantial heterogeneity in the study design or outcomes was detected.

**Conclusions:**

Identifying the optimal parameters for gene editing to enhance engraftment efficiency may provide crucial insights for designing future clinical trials and advancing the therapeutic application of CRISPR-Cas9 edited HSPCs.

## Introduction

Genetic diseases from mutations in hematopoietic cell lineages, such as sickle cell disease, β-thalassemia, and severe combined immunodeficiency (SCID), pose significant clinical challenges with limited curative options available. Hematopoietic stem cell transplantation (HSCT) offers a potential cure for numerous inherited conditions, including thalassemia, bone marrow (BM) failure syndromes, primary immunodeficiencies, Gaucher disease, Hunter syndrome, or various mucopolysaccharidoses [1]. In HSCT, hematopoietic stem cells are intravenously infused to restore blood cell production. Transplants can be autologous (patient’s own cells), allogeneic (from a donor), or syngeneic (from an identical twin). Cell sources include BM, peripheral blood (PB), umbilical cord blood (UCB), and fetal liver, each with distinct advantages and limitations. Successful transplantation depends on donor availability and conditioning regimens involving chemotherapy, radiation, or monoclonal antibodies [2]. Currently, HSCT is used to treat over 70 diseases, with the number growing rapidly. According to the Worldwide Network for Blood and Marrow Transplantation, approximately 90,000 HSCTs are performed annually worldwide, of which 53% are autologous and 47% are allogeneic [3]. Advances in conditioning strategies, infection control, and graft-versus-host disease (GVHD) management have improved patient outcomes despite persistent donor matching challenges [4].

Gene therapy has emerged as a transformative approach for treating genetic disorders, particularly those involving hematopoietic dysfunctions. *Ex vivo* gene transfer into hematopoietic stem and progenitor cells (HSPCs) is a pivotal strategy for diseases amenable to BM transplantation [5]. Hematopoietic gene therapy has pioneered the initial clinical proof of concept for treating SCID in human patients [6]. Recent advancements in genome-editing technologies such as transcription activator-like effector nucleases (TALENs), zinc-finger nucleases (ZFNs), homing endonucleases, and especially CRISPR-Cas9 (clustered regularly interspaced short palindromic repeats and CRISPR-associated protein 9) facilitate precise, efficient, and versatile modifications of cellular genomes [7]. When HSPCs are edited *ex vivo* and subsequently transplanted, they can achieve long-term engraftment and confer systemic therapeutic benefits [8]. Currently, several hematopoietic gene therapy products are available, including the first CRISPR/Cas9-based therapy using *ex vivo* edited HSPCs for treating sickle cell disease [9].

The success of hematopoietic gene therapy has historically relied on myeloablative conditioning, which enhances multilineage HSPC engraftment and enables sustained gene correction [10]. Over the years, mobilized PB HSPCs have emerged as the standard cell source for *ex vivo* therapies, demonstrating long-term multilineage engraftment comparable to that of BM-derived HSPCs [11]. Gene transfer vectors play a crucial role in ensuring the safety and efficacy of these therapies [12]. Recent advances, including mRNA-based and non-viral *in vivo* approaches, offer the potential for non-genotoxic conditioning, thereby obviating the need for chemotherapy by facilitating the selection of HSPCs with a high degree of gene modification [13]. Despite these advancements, a significant challenge persists in achieving a safe gene dose that ensures an effective therapeutic level *in vivo* [14]. Preclinical evaluation of gene-edited HSPCs in animal models is essential to assess engraftment efficiency, long-term persistence, and functional correction of target diseases [15]. Engraftment reflects the ability of edited HSPCs to repopulate the BM and sustain multilineage hematopoiesis [16]. Despite numerous studies demonstrating CRISPR/Cas9-mediated gene editing in HSPCs, variability in design, delivery, efficiency, and outcome metrics makes it challenging to synthesize the data. This systematic review and meta-analysis consolidates current preclinical evidence to quantitatively assess the engraftment potential of CRISPR/Cas9-edited HSPCs in curing monogenic hematologic disorders.

## Materials and Methods

### Study design

We conducted this study in accordance with the protocols established in previously published systematic reviews and meta-analysis, adhering to the Preferred Reporting Items for Systematic Reviews and Meta-Analyses (PRISMA) guidelines [17].

### Eligibility criteria

We established specific inclusion and exclusion criteria for the results obtained from the literature search and screened the studies accordingly. The inclusion criteria were as follows: 1) original and full-length articles; 2) preclinical animal models transplanted with CRISPR/Cas9-edited HSPCs; 3) non-edited HSPCs; 4) studies using laboratory mice; and 5) articles published in English.

The exclusion criteria were as follows: 1) review articles; 2) clinical reports and/or trials; 3) studies involving non-CRISPR gene editing techniques; and 5) studies that failed to provide the required information. There were no restrictions regarding species, age, gender, duration of transplantation, and administration of CRISPR/Cas9-edited HSPCs.

### Search strategy

**Figure 1:**
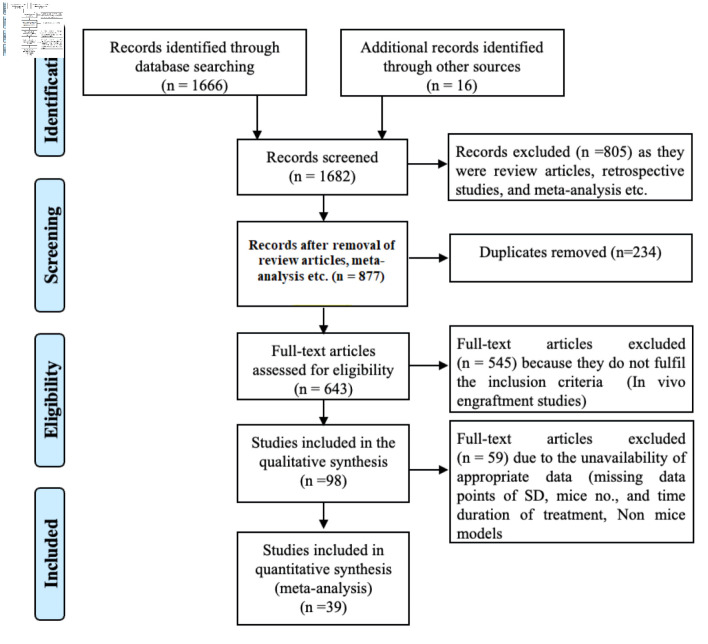
Preferred Reporting Items for Systematic Reviews and Meta-Analyses (PRISMA) flowchart for the systematic literature search and study inclusion.

Three independent researchers (SY, PKS, and AK) conducted a comprehensive literature search using PubMed Medline, Web of Science, and Google Scholar to identify studies that assessed the effect of CRISPR/Cas9-mediated gene editing of HSPCs on transplantation in mice to correct genetic diseases. The search strategy employed various terms, including (“CRISPR-Cas9”) AND (“gene therapy” OR “gene editing”) AND (“Hematologic disorder” OR “Hemoglobinopathy” OR “Hemoglobinopathies” OR “sickle cell disease” OR “sickle cell anemia” OR “SCD” OR “hematopoietic progenitor stem cell” OR “HSPC” OR “hematopoietic stem cell” OR “HSC” OR “thalassemia” OR “beta thalassemia” OR “alpha thalassemia” OR “thalassemia major” OR “thalassemia minor” OR “animal engraftment” OR “mouse engraftment” OR “HSC engraftment” OR “HPSC engraftment” OR “stem cell transplantation” OR “bone marrow transplantation” OR “peripheral blood stem cell transplantation” OR “hematopoietic stem cell transplantation” OR “HSC transplantation” OR “hematopoietic progenitor stem cell transplantation” OR “HPSC transplantation”). We manually reviewed the references cited in the relevant articles. The results of the literature search are presented in a PRISMA flow chart (Fig. 1), showing the study selection process.

### Data extraction

Three investigators (SY, BC, and BA) independently screened the literature, resolving any disagreements through discussions with additional authors (AK, SY, and PKS). Data were quantitatively extracted from bar plots in each article using the WebPlotDigitizer program and from the tables, subsequently organized in a Microsoft Excel spreadsheet (Windows 10 edition; Microsoft Corporation, Lisbon, Portugal) to record species and strains, the number of animals/groups, model cell lines, route of administration of cell used for engraftment in animal models.

### Quantitative data analysis

Pooled data analysis was conducted using Jamovi Software Version 2.6.45.0, with Hedge’s g selected as the “effect size” metric [18]. Heterogeneity among studies was evaluated using Cochran’s Q test and the heterogeneity index (I^2^). A significance threshold of P < 0.10 was applied because of the sensitivity of the test. The quantitative assessment of heterogeneity was based on the I^2^ scale: low (< 25%), moderate (50–75%), and high (> 75%). The fixed or random effects model was chosen to compute the pooled effect size based on the level of heterogeneity. When the I^2^ value was ≤ 50%, fixed effect models were applied; however, data with an I^2^ value > 50% were analyzed using the random effect model. Subgroup analysis was conducted based on mouse strains, knock-in or knockout strategy, mode of transplantation, source of HSPC, CRISPR system, homology-directed repair (HDR) template, and targeted disease.

### Sensitivity analysis

Sensitivity analysis was conducted by systematically excluding one study at a time to assess its impact on the pooled effect size estimate. This approach was used to evaluate the influence of individual studies on overall findings.

### Publication bias analysis

We conducted an analysis of publication bias by qualitatively examining the asymmetry in the funnel plot and quantitatively based on Egger’s intercept and Begg and Mazumdar rank test.

Institutional review board approval and ethical compliance with human or animal studies were not applicable for this study.

## Results

### Study design and parameters measured

A comprehensive literature search yielded 1,682 potential articles, of which 39 studies were included. These studies targeted 18 different gene loci, distributed as follows: *HBB* (n = 8), *HBG* (n = 5), *CD33* (n = 4), *CCR5*, *ELANE*, and *BCL11* (n = 3 each), *CYBB*, CD45 (n = 2 each), and *TET2*, Cohesin, *ITGB2*, CD40L, *FXN*, *FOXP3*, *MAGT1*, *PKLR*, *WAS*, *IL2RG*, and *BTK* (n = 1 each). All these studies employed Cas9 as a nuclease delivered in the form of ribonucleoprotein (ribonucleoprotein complex (RNP), n = 31), RNA (n = 4), plasmid (n = 3), and virus (n = 1) via electroporation (n = 36), transfection (n = 2), and nanoparticles and transduction (n = 1 each). These articles utilized Cas9 for genomic modifications through knockout and knock-in (n = 19 each), while one study performed base editing. During gene editing via knock-in, HDR templates were delivered as single-strand oligonucleotide (ssODNs, n = 6) and through adeno-associated virus (AAV, n = 15), where two studies used both, as indicated in Table 1 [19–57]. The literature review and study screening results are shown in the PRISMA flow diagram in Figure 1.

**Table 1:** Characteristic Summary of the Preclinical Studies Included in the Metanalysis

Author and year	Goal of gene editing	Cas9 delivery system	Donor template delivery system	Targeted gene	Tissue source of hHSPCs	Mice strain	Mode of transplantation	Number of cells injected	Follow-up duration after transplantation
Xu et al, 2017 [19]	Knockout	Plasmid	No donor template	*CCR5*	Fetal liver	NSG	Intrahepatic	1 × 10^6^	12 weeks
Tothova et al, 2017 [20]	Knockout	Plasmid	No donor template	*TET2*/Cohesin	PB	NSGS	Retro-orbital	0.2–0.5 × 10^6^	22 weeks
De Ravin et al, 2017 [21]	Knock-in	mRNA/gRNA	ssODN	*CYBB*	PB	NSG	Tail vein	1–3 × 10^6^	20 weeks
Yen et al, 2018 [22]	Knockout	RNP	No donor template	*HBB*	BM	NSG	Tail vein	0.7 × 10^6^	12–20 weeks
Kim et al, 2018 [23]	Knockout	RNP	No donor template	*CD33*	PB	NSG	Tail vein	0.1–0.5 × 10^6^	16 weeks
Pattabhi et al, 2019 [24]	Knock-in	RNP	AAV6/ssODN	*HBB*	PB	NBSGW	Tail vein	2 × 10^6^	14 weeks
Borot et al, 2019 [25]	Knockout	RNP	No donor template	*CD33*	BM	NSGSGM3	Tail vein	0.5–1 × 10^6^	9–21 weeks
Romero et al, 2019 [26]	Knock-in	RNP	AAV6/ssODN	*HBB*	PB	NSG	Retro-orbital	1–1.3 × 10^6^	16 weeks
Metais et al, 2019 [27]	Knockout	RNP	No donor template	*HBG1/HBG2*	PB	NBSGW	Tail vein	1 × 10^6^	16–17 weeks
Bloomer et al, 2021 [28]	Knock-in	RNP	AAV6	*ITGB2*	PB	NSG	Tail vein	0.3 × 10^6^	18 weeks
Yudovich et al, 2020 [29]	Knockout	RNP	No donor template	CD45	UCB	NSG	Tail vein	0.1 × 10^6^	12 weeks
Tran et al., 2020 [30]	Knock-in	RNP	AAV6	*ELANE*	PB	NOG-XL	Tail vein	0.2 × 10^6^	12–17 weeks
Rai et al, 2020 [31]	Knock-in	RNP	AAV6	*ELANE*	PB	NSG	Tail vein	0.5 × 10^6^	14 weeks
Rocca et al, 2020 [32]	Knockout	RNP	No donor template	*FXN*	PB	NSG	Intrahepatic	1 × 10^6^	12 weeks
Goodwin et al, 2020 [33]	Knock-in	RNP	AAV6	*FOXP3*	UCB	NSGSGM3	Intrahepatic	0.15–1 × 10^6^	8–14 weeks
Weber et al, 2020 [34]	Knockout	RNP	No donor template	*HBG1/HBG2*	PB	NSG	Intraperitoneal	1 × 10^6^	16 weeks
Brault et al, 2021 [35]	Knock-in	mRNA/gRNA	AAV	*MAGT1*	PB	NSGS	Intrahepatic	1–1.5 × 106^6^	16 weeks
Sweeney et al, 2021 [36]	Knock-in	RNP	AAV6	*CYBB*	PB	NSG	Tail vein	1–2 × 10^6^	12 weeks
Uchida et al, 2021 [37]	Knock-in	RNP	ssDNA	*HBB*	PB	NBSGW	Tail Vein	0.4–0.5 × 10^6^	12–16 weeks
Psatha et al, 2021 [38]	Knockout	RNP	No donor template	*BCL11*	PB	NBSGW	Unknown	2 × 10^6^	16 weeks
Fananas-Baquero et al, 2021 [39]	Knock-in	RNP	AAV6	*PKLR*	UCB	NSG	Tail vein	1 × 10^6^	13 weeks
Li et al, 2021 [40]	Knockout	HdAd	No donor template	*HBG2*	PB	NSG	Tail vein	0.5 × 106^6^	8 weeks
Samuelson et al, 2021 [41]	Knockout	RNP	No donor template	*HBG/BCL11*	PB	NSG	Tail vein	1 × 10^6^	12–23 weeks
El-Kharrag et al, 2022 [42]	Knockout	RNP	No donor template	*CD33*	PB	NSG	Tail vein	0.5 × 10^6^	20 weeks
Karrupusamy et al, 2022 [43]	Knockout	RNP	No donor template	*CCR5*	PB	NBSGW	Tail vein	0.09 × 10^6^	16 weeks
Venkatesan et al,2023 [44]	Knockout	RNP	No donor template	PRR β-globin	PB	NBSGW	Tail vein	0.5–0.6 × 10^6^	16 weeks
Rai et al, 2023 [45]	Knock-in	RNP	AAV6	*WAS*	PB	NSG	Tail vein	0.5 × 10^6^	8–16 weeks
Wellhausen et al,2023 [46]	Knockout	RNP	No donor template	CD45	PB	NSG	Tail vein	0.5 × 10^6^	12 weeks
Lydeard et al, 2023 [47]	Knockout	RNP	No donor template	*CD33*	PB	NSG	Tail vein	0.1–1 × 10^6^	16 weeks
Hardouin et al, 2023 [48]	Editing	mRNA/gRNA	No donor template	*HBB*	PB	NBSGW	Retro-orbital	0.26 × 10^6^	16 weeks
Brault et al, 2023 [49]	Knock-in	mRNA/gRNA	AAV6	*IL2RG*	PB	NSGS	Intrahepatic	1–1.5 × 10^6^	12–16 weeks
Frati et al, 2024 [50]	Knockout	RNP	No donor template	γ-globin promoter	PB	NSG	Retro-orbital	0.5–2 × 10^6^	16 weeks
Bahal et al, 2024 [51]	Knock-in	RNP	AAV6	*BTK*	PB	NSG	Tail vein	2 × 10^6^	15 weeks
Demirci et al, 2024 [52]	Knockout	RNP	No donor template	*BCL11*	PB	NBSGW	Tail vein	0.3 × 10^6^	24 weeks
Nasri et al, 2024 [53]	Knock-in	RNP	ssODN	*ELANE*	BM	NSG	Intrafemoral	0.1 × 10^6^	16 weeks
Pugliano et al, 2024 [54]	Knock-in	RNP	AAV6	CD40L	PB	NBSGW	Retro-orbital	1.5–2 × 10^6^	12–16 weeks
Dudek et al, 2024 [55]	Knock-in	RNP	AAV6	CCR5	PB	NSGSGM3	Retro-orbital	1 × 10^6^	14 weeks
Park et al, 2019 [56]	Knock-in	Plasmid	ssODN	HBB	PB	NSG	Intrafemoral	0.5 × 10^6^	19 weeks
Dever et al., 2016 [57]	Knock-in	RNP	AAV6	HBB	PB	NSG	Tail vein	0.4–0.7 × 10^6^	Unknown

hHSPCs: human hematopoietic stem and progenitor cells; mRNA: messenger ribonucleic acid; gRNA: guide RNA; RNP: ribonucleoprotein complex; AAV6: adeno-associated virus type 6; ssODN: single-stranded oligodeoxynucleotides; PB: peripheral blood; BM: bone marrow; UCB: umbilical cord blood; CCR5: C-C chemokine receptor type 5; TET2: Tet methylcytosine deoxygenase 2; CYBB: cytochrome b-245 beta chain; HBB: hemoglobin subunit beta; CD: cluster of differentiation; HBG: hemoglobin subunit gamma; ITGB2: integrin beta subunit 2; ELANE: neutrophil elastase; FXN: frataxin; FOXP3: forkhead box P3; MAGT1: magnesium transporter 1; BCL11: B-cell lymphoma/leukemia 11; PKLR: pyruvate kinase L/R; PRR: putative repressor region; WAS: Wiskott Aldrich syndrome; IL2RG: interleukin 2 receptor subunit gamma; BTK: Bruton’s tyrosine kinase; NSG: non-obese diabetic (NOD) Scid gamma.

The majority of the studies utilized mobilized PB stem cells (n = 32), while some used UCB stem cells and BM-derived HSPCs (n = 3 each). One study utilized fetal liver derived stem cells. Mobilized PB HSPCs and BM-derived HSPCs were isolated from patient samples or healthy individuals. These cells were purified using antibodies against the CD34 epitope. Purified CD34^+^ cells were suspended in 1 × phosphate-buffered saline (PBS) or 1% saline at various concentrations for injection into mice. The total number of cells injected per mouse ranged from 0.09 × 10^6^ to 3 × 10^6^ cells. Specifically, 20 studies used 0.09–0.5 × 10^6^ cells, 18 studies used 0.5–1 × 10^6^ cells, seven studies used 1–1.5 × 10^6^ cells, and six studies used 1.5–3 × 10^6^ cells (Table 1) [19–57]. Some studies have employed varying cell quantities across different experiments.

We have provided details of the mice models and transplantation techniques used in all investigations (Table 1) [19–57]. Most of the studies utilized the tail vein as the preferred transplantation route (24 studies). Alternative methods included retro-orbital (six studies), intraperitoneal (one study), intrahepatic (five studies), and intra-femoral (two studies), while one publication did not provide the route of transplantation [38]. All of these studies utilized four different mouse strains, NSG (23 studies), NSGS, NSGSGM3 (three studies each), NBSGW (nine studies), and one study used the NOG-EXL mouse strain. The follow-up period for engraftment analysis varied from 8 weeks to 24 weeks, where most of the experiments were terminated between 12 and 16 weeks (25 studies), while other studies monitored the engraftment within 8–12 weeks (eight studies), 16–20 weeks (seven studies) and 20–24 weeks (four studies), as detailed in Table 1 [19–57].

We measured the engraftment potential of gene edited versus unedited cells in the BM, spleen, thymus and PB (Table 2). Human cell chimerism in all transplanted mice was analyzed by flow cytometry using CD45 as a marker, while two studies used human leukocyte antigen ABC (HLA-ABC) for quantification because they had deleted the CD45 locus for their study.

**Table 2:** Summary of the Pooled Data and Subgroup Analysis of Various Parameters of the Study

Parameter	Groups	Subgroups	Test of heterogeneity			Test model	Types of association				Significance
			Q	P	I^2^ (%)		Hedge’s g	Lower limit	Upper limit	P value	
Bone marrow engraftment	Gene editing	Pooled	28.446	1.000	0.000	Fixed	–0.160	–0.292	–0.028	0.018	Significant
		Knock-in	5.681	1.000	0.000	Fixed	0.103	–0.092	0.297	0.302	NS
		Knockout	21.855	0.977	0.000	Fixed	–0.218	–0.401	–0.035	0.020	Significant
	CRISPR-Cas9 system	RNA	0.448	0.998	0.000	Fixed	–0.032	–0.412	0.348	0.869	NS
		RNP	27.505	1.000	0.000	Fixed	–0.177	–0.318	–0.037	0.014	Significant
	Mice strain	NSG	19.312	0.993	0.000	Fixed	–0.238	–0.426	–0.051	0.013	Significant
		NSGS	0.753	0.686	0.000	Fixed	0.324	–0.524	1.172	0.454	NS
		NBSGW	0.885	1.000	0.000	Fixed	–0.055	–0.287	0.176	0.639	NS
		NSGSGM3	4.479	0.812	0.000	Fixed	–0.286	–0.753	0.181	0.229	NS
	Route of transplantation	Tail vein	9.191	1.000	0.000	Fixed	–0.150	–0.314	0.013	0.072	NS
		Intrahepatic	1.290	0.863	0.000	Fixed	–0.011	–0.424	0.402	0.958	NS
		Retro-orbital	14.159	0.166	29.37	Fixed	–0.502	–0.894	–0.109	0.012	Significant
		Intraperitoneal	0.788	0.852	0.000	Fixed	–0.375	–1.078	0.328	0.296	NS
	Source of HSPC	PB	23.709	1.000	0.000	Fixed	–0.166	–0.305	–0.027	0.019	Significant
		BM	3.813	0.577	0.000	Fixed	–0.407	–0.909	0.095	0.112	NS
		UCB	1.634	0.897	0.000	Fixed	0.008	–0.552	0.569	0.976	NS
	Disease targeted	HIV	0.668	0.955	0.000	Fixed	–0.394	–0.987	0.199	0.193	NS
		IMD	3.392	1.000	0.000	Fixed	–0.101	–0.335	0.133	0.397	NS
		HGP	16.408	0.945	0.000	Fixed	–0.199	–0.407	0.009	0.061	NS
		SCN	0.530	0.912	0.000	Fixed	0.092	–0.570	0.755	0.785	NS
		Leukemia	5.806	0.926	0.000	Fixed	–0.183	–0.501	0.134	0.258	NS
	HDR template	AAV6	4.336	1.000	0.000	Fixed	–0.141	–0.350	0.068	0.187	NS
		ssODN	0.387	0.999	0.000	Fixed	0.143	–0.386	0.673	0.596	NS
Spleen engraftment	Gene editing	Pooled	20.908	0.587	0.000	Fixed	–0.307	–0.527	–0.087	0.006	Significant
		Knock-in	7.043	0.532	0.000	Fixed	0.012	–0.269	0.293	0.933	NS
		Knockout	7.881	0.851	0.000	Fixed	–0.663	–1.000	–0.327	< 0.001	Significant
	CRISPR-Cas9 system	RNA	0.997	0.608	0.000	Fixed	0.374	–0.054	0.802	0.087	NS
		RNP	12.694	0.890	0.000	Fixed	–0.457	–0.702	–0.212	< 0.001	Significant
	Mice strain	NSG	5.382	0.864	0.000	Fixed	–0.764	–1.145	–0.383	< 0.001	Significant
		NSGS	NA	NA	NA	NA	NA	NA	NA	NA	NA
		NBSGW	2.518	0.866	0.000	Fixed	–0.147	–0.495	0.200	0.405	NS
		NSGSGM3	0.291	0.962	0.000	Fixed	–0.484	–1.188	0.221	0.178	NS
	Route of transplantation	Tail vein	6.600	0.472	0.000	Fixed	–0.428	–0.837	–0.019	0.040	Significant
		Intrahepatic	NA	NA	NA	NA	NA	NA	NA	NA	NA
		Retro-orbital	3.917	0.917	0.000	Fixed	–0.330	–0.657	–0.004	0.047	Significant
		Intraperitoneal	0.685	0.877	0.000	Fixed	–0.983	–1.721	–0.245	0.009	Significant
	Source of HSPC	PB	NA	NA	NA	NA	NA	NA	NA	NA	NA
		BM	NA	NA	NA	NA	NA	NA	NA	NA	NA
		UCB	NA	NA	NA	NA	NA	NA	NA	NA	NA
	Disease targeted	HIV	0.315	0.989	0.000	Fixed	–0.434	–1.027	0.159	0.152	NS
		IMD	2.139	0.343	6.490	Fixed	0.235	–0.122	0.592	0.196	NS
		HGP	6.797	0.871	0.000	Fixed	–0.479	–0.793	–0.166	0.003	Significant
		SCN	NA	NA	NA	NA	NA	NA	NA	NA	NA
		Leukemia	2.783	0.249	28.140	Fixed	–1.020	–1.919	–0.121	0.026	Significant
	HDR template	AAV6	7.043	0.532	0.000	Fixed	0.012	–0.269	0.293	0.933	NS
		ssODN	NA	NA	NA	NA	NA	NA	NA	NA	NA
Thymus engraftment	Gene editing	Pooled	4.565	0.918	0	Fixed	–0.097	–0.435	0.240	0.572	NS
		Knock-in	3.772	0.287	20.46	Fixed	0.159	–0.174	0.493	0.349	NS
		Knockout	4.009	0.676	0.000	Fixed	–0.087	–0.627	0.452	0.752	NS
	CRISPR-Cas9 system	RNA	3.744	0.287	20.500	Fixed	0.159	–0.174	0.493	0.348	NS
		RNP	4.010	0.675	0.000	Fixed	–0.087	–0.627	0.452	0.751	NS
	Mice strain	NSG	4.288	0.509	0.000	Fixed	–0.153	–0.557	0.251	0.459	NS
		NSGS	1.313	0.859	0.000	Fixed	0.328	–0.070	0.727	0.106	NS
		NBSGW	NA	NA	NA	NA	NA	NA	NA	NA	NA
		NSGSGM3	NA	NA	NA	NA	NA	NA	NA	NA	NA
	Route of transplantation	Tail vein	3.866	0.145	48.260	Fixed	–0.216	–1.075	0.643	0.622	NS
		Intrahepatic	3.847	0.279	22.010	Fixed	0.157	–0.177	0.490	0.357	NS
		Retro-orbital	NA	NA	NA	NA	NA	NA	NA	NA	NA
		Intraperitoneal	0.000	1.000	0.000	Fixed	–0.003	–0.687	0.680	0.993	NS
	Source of HSPC	PB	NA	NA	NA	NA	NA	NA	NA	NA	NA
		BM	NA	NA	NA	NA	NA	NA	NA	NA	NA
		UCB	NA	NA	NA	NA	NA	NA	NA	NA	NA
	Disease targeted	HIV	NA	NA	NA	NA	NA	NA	NA	NA	NA
		IMD	3.773	0.287	20.500	Fixed	0.159	–0.174	0.493	0.349	NS
		HGP	0.040	1.000	0.000	Fixed	0.029	–0.583	0.643	0.924	NS
		SCN	NA	NA	NA	NA	NA	NA	NA	NA	NA
		Leukemia	3.718	0.156	46.210	Fixed	–0.216	–1.074	0.642	0.621	NS
	HDR template	AAV6	3.774	0.287	20.500	Fixed	0.159	–0.174	0.493	0.348	NS
		ssODN	NA	NA	NA	NA	NA	NA	NA	NA	NA
Peripheral blood engraftment	Gene editing	Pooled	12.463	1.000	0.000	Fixed	–0.270	–0.464	–0.075	0.007	Significant
		Knock-in	16.622	0.992	0.000	Fixed	–0.128	–0.307	0.051	0.162	NS
		Knockout	11.536	1.000	0.000	Fixed	–0.231	–0.442	–0.020	0.032	Significant
	CRISPR-Cas9 system	RNA	0.721	0.998	0.000	Fixed	0.348	0.046	0.649	0.024	Significant
		RNP	20.832	1.000	0.000	Fixed	–0.242	–0.388	–0.096	0.001	Significant
	Mice strain	NSG	NA	NA	NA	NA	NA	NA	NA	NA	NA
		NSGS	3.719	0.445	0.000	Fixed	0.313	0.011	0.615	0.042	Significant
		NBSGW	5.341	0.989	0.000	Fixed	–0.233	–0.476	0.010	0.060	NS
		NSGSGM3	0.157	0.984	0.000	Fixed	0.120	–0.481	0.721	0.696	NS
	Route of transplantation	Tail vein	9.513	1.000	0.000	Fixed	–0.207	–0.394	–0.020	0.030	Significant
		Intrahepatic	3.731	0.881	0.000	Fixed	0.224	–0.040	0.488	0.096	NS
		Retro-orbital	5.671	0.579	0.000	Fixed	–0.247	–0.533	0.039	0.090	NS
		Intraperitoneal	0.127	0.988	0.000	Fixed	–0.887	–1.614	–0.160	0.017	Significant
	Source of HSPC	PB	28.135	1.000	0.000	Fixed	–0.172	–0.315	–0.029	0.018	Significant
		BM	0.035	0.983	0.000	Fixed	–0.055	–0.741	0.630	0.875	NS
		UCB	0.157	0.984	0.000	Fixed	0.120	–0.481	0.721	0.696	NS
	Disease targeted	HIV	NA	NA	NA	NA	NA	NA	NA	NA	NA
		IMD	11.636	0.865	0.000	Fixed	–0.128	–0.334	0.078	0.224	NS
		HGP	11.512	1.000	0.000	Fixed	–0.224	–0.432	–0.016	0.035	Significant
		SCN	0.878	0.831	0.000	Fixed	–0.125	–0.789	0.540	0.713	NS
		Leukemia	NA	NA	NA	NA	NA	NA	NA	NA	NA
	HDR template	AAV6	15.384	0.698	0.000	Fixed	–0.082	–0.267	0.103	0.384	NS
		ssODN	3.198	0.994	0.000	Fixed	0.095	–0.359	0.549	0.682	NS

CRISPR-Cas9: clustered regularly interspaced short palindromic repeats and CRISPR-associated protein 9; HDR: homology-directed repair; PB: peripheral blood; RNP: ribonucleoprotein complex; BM: bone marrow; UCB: umbilical cord blood; HIV: human immunodeficiency virus; IMD: immune mediated disease; HGP: hemoglobinopathy; SCN: severe congenital neutropenia; AAV6: adeno-associated virus type 6; ssODN: single-strand oligonucleotide; NA: data not available for analysis; HSPC: hematopoietic stem progenitor cell; NS: not significant.

### Effect of CRISPR/Cas9-edited HSPCs on engraftment potential in BM, PB, spleen, and thymus

**Figure 2:**
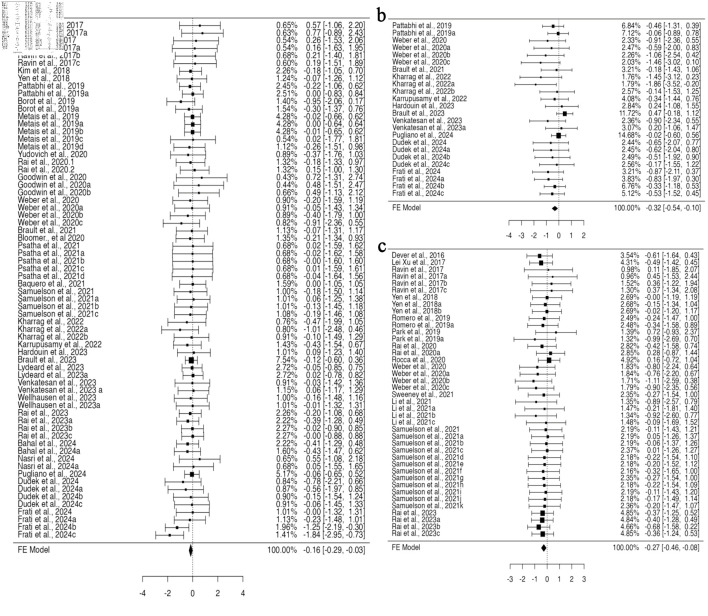
CRISPR-Cas9 gene editing negatively impacts the ability of hematopoietic stem and progenitor cells to engraft *in vivo*, as measured by fluorescent-activated cell sorting. (a) Analysis of bone marrow engraftment comprised a total of 68 datasets. Most estimates were negative (68%), with observed standardized mean differences ranging from –1.8391 to 0.7740. (b) Analysis of spleen engraftment comprised a total of 24 datasets. Most estimates were negative (88%), and the observed standardized mean differences varied from –1.8629 to 0.4730. (c) Analysis of peripheral blood engraftment comprised a total of 41 datasets. The majority of estimates (78%) were negative, with observed standardized mean differences ranging from –1.1051 to 0.7224. CRISPR-Cas9: clustered regularly interspaced short palindromic repeats and CRISPR-associated protein 9.

In a pooled group analysis following the fixed effect model, a significant difference was observed in the mean difference between gene-edited and unedited cells. Twenty-nine studies using BM for their engraftment analysis with 68 datasets were incorporated into the pooled analysis (Supplementary Material 1, jh.elmerpub.com). The fixed effect model analysis demonstrated a significant reduction in the engraftment of gene-edited cells in the BM (95% confidence interval (CI), –0.292 to –0.028; P = 0.018) (Fig. 2a). The findings of the studies were unbiased, as neither the rank correlation nor the regression test indicated any funnel plot asymmetry (P = 0.1373 and P = 0.7823, respectively) (Supplementary Material 2A, jh.elmerpub.com). Additionally, 11 studies presented 24 datasets for spleen engraftment (Supplementary Material 3, jh.elmerpub.com), and 24 studies presented 41 datasets for PB engraftment (Supplementary Material 4, jh.elmerpub.com). The fixed effect model analysis revealed a significant decline in the engraftment of gene-edited HSPCs in the spleen and PB (95% CI, –0.542 to –0.097; P = 0.005; 95% CI, –0.464 to –0.075; P = 0.007, respectively) (Fig. 2b, c). However, the spleen data were homogenous in nature (Tau^2^ = 0.0316; I^2^ = 9.07%) and biased, as both the rank correlation and regression tests indicated potential funnel plot asymmetry (P < 0.0001 and P = 0.0016, respectively) (Supplementary Material 2B, jh.elmerpub.com). Conversely, the PB data were homogeneous and unbiased, as evidenced by funnel plot symmetry (correlation P = 0.9023 and regression P = 0.7030) (Supplementary Material 2C, jh.elmerpub.com). Furthermore, the data presented for thymus engraftment (five studies, 11 datasets) indicate no discernible difference in engraftment capacity between gene-edited and unedited HSPCs (Supplementary Material 5, jh.elmerpub.com).

### Effect of CRSIPR/Cas9 gene editing strategy through knock-in or knockout on engraftment potential

**Figure 3:**
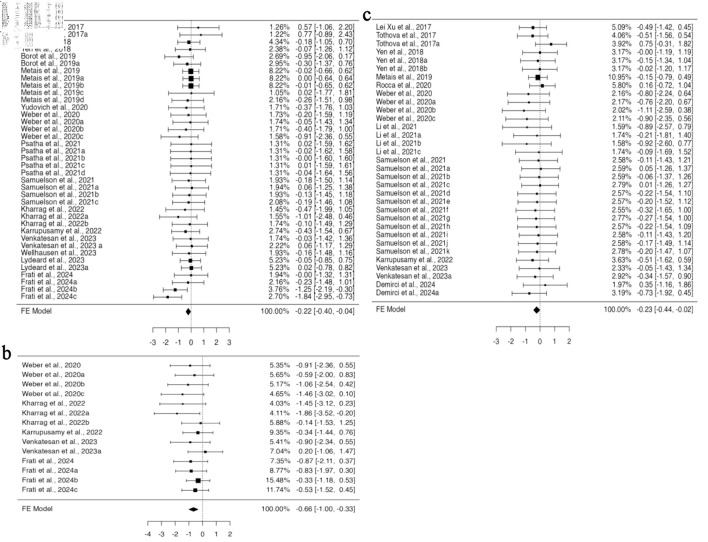
The *in vivo* engraftment potential of human CD34^+^ HSPCs is impacted by the gene knockout approach used for gene editing. (a) Analysis of bone marrow engraftment included 38 datasets. The majority of estimates (76%) were negative, with observed standardized mean differences ranging from –1.8391 to 0.7740. (b) Analysis of spleen engraftment comprised a total of 14 datasets, of which 93% of the estimates were negative, with the observed standardized mean differences ranging from –1.8629 to 0.2046. (c) Analysis of peripheral blood engraftment comprised a total of 33 datasets. Most estimates (85%) were negative, with the observed standardized mean differences ranging from –1.1051 to 0.7534. HSPCs: hematopoietic stem and progenitor cells.

Gene editing encompasses both the insertion of DNA (knock-in) and the deletion of genes (knockout), and both methodologies were reported in the included studies. HSPCs genetically modified through knock-in did not exhibit a significant difference in the standardized mean difference compared to non-edited cells for the data provided across all analyzed organs. Conversely, knockout HSPCs demonstrate significant variations in engraftment efficacy in specific organs. For BM engraftment, 13 studies provided 28 datasets for knock-in, and 15 studies provided 38 datasets for knockout HSPCs. A fixed effect model applied to knockout HSPCs revealed a statistically significant standardized mean difference (95% CI, –0.401 to –0.035; P = 0.020) (Fig. 3a), suggesting a decrease in the engraftment of knockout HSPCs in the BM; however, the data were homogenous without any funnel plot asymmetry (Supplementary Material 6A, jh.elmerpub.com). For PB engraftment, 11 studies provided 33 datasets for the knockout, and 34 datasets from 13 studies for knock-in HSPCs. There was no heterogeneity; however, some bias in the data was determined based on Tau^2^ and funnel plot asymmetry (Supplementary Material 6C, jh.elmerpub.com). Using a fixed effect model, the data analysis revealed a significant difference in the standardized mean difference measure (95% CI, –0.442 to –0.020; P = 0.032) (Fig. 3c). This concedes the lower engraftment of knockout HSPCs in the PB compared to unedited counterparts. Among the 12 articles providing data on spleen engraftment, 14 experimental studies contributed data on gene knockout and nine on knock-in. The available data exhibited no heterogeneity but were slightly biased, as revealed by the Tau^2^ and funnel plot asymmetry (Supplementary Material 6B, jh.elmerpub.com). The fixed effect model analysis concedes a highly significant standardized mean difference for the engraftment of knockout HSPCs in the spleen compared to non-edited ones (95% CI, –1.00 to –0.327; P < 0.001) (Fig. 3b).

### The consequences of CRISPR-Cas9 system used for gene editing on the engraftment of gene-edited HSPCs

**Figure 4:**
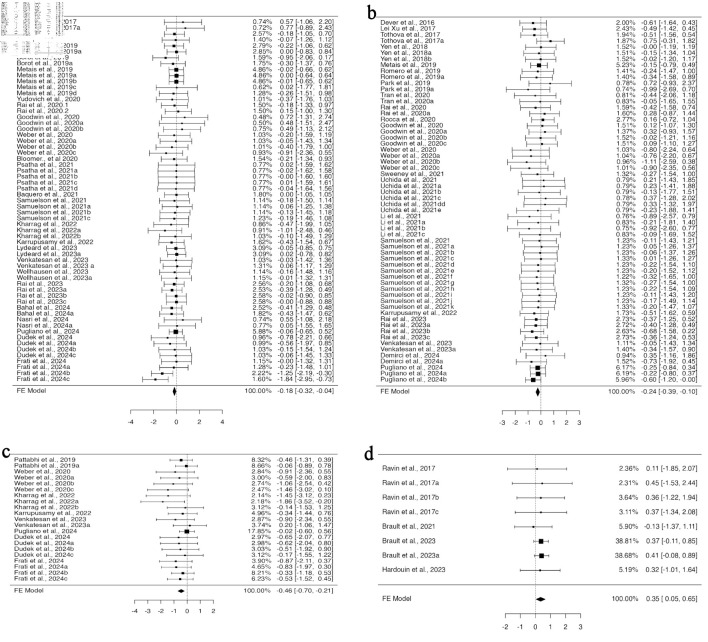
CRISPR-Cas9-mediated gene editing using RNP complex affects the *in vivo* engraftment of human HSPCs in mice model. (a) The analysis of bone marrow engraftment comprised 61 datasets that used the RNP version of the CRISPR-Cas9 system. The majority of estimates (72%), with observed standardized mean differences ranging from –1.8391 to 0.7740, were negative. (b) The study of peripheral blood engraftment included 60 datasets that used the RNP version of the CRISPR-Cas9 technology. A significant proportion of estimates (77%), with observed standardized mean differences ranging from –1.1051 to 0.7534, were negative. (c) Twenty-one datasets in all that used the RNP version of the CRISPR-Cas9 technology were analyzed for spleen engraftment. The observed standardized mean differences were mostly negative (95%), with a range of –1.8629 to 0.2046. (d)The investigation of peripheral blood engraftment comprised a total of eight datasets that used the CRISPR-Cas9 system in RNA form. The majority of estimates (88%), with observed standardized mean differences ranging from –0.1343 to 0.4505, were positive. CRISPR-Cas9: clustered regularly interspaced short palindromic repeats and CRISPR-associated protein 9; HSPCs: hematopoietic stem and progenitor cells; RNP: ribonucleoprotein complex.

To perform gene editing, guide RNA and CRISPR-Cas9 can be introduced into cells as RNA, RNP, or in the form of plasmids. Among all the studies included in the BM engraftment analysis, 25 articles provided 61 investigations with data for RNP, while seven datasets for RNA from four studies, for gRNA/CRISPR-Cas9 delivery system. No heterogeneity was found in the data for RNP (Supplementary Material 7A, jh.elmerpub.com); therefore, the fixed effect model analysis suggested a significant variation in the standardized mean difference (95% CI, –0.318 to –0.037; P = 0.014) (Fig. 4a). This signifies decreased engraftment of gene-edited HSPCs in the BM when using RNP as a delivery system for CRISPR-Cas9, while no effect was observed when using them in RNA form. For the investigation of spleen engraftment of gene-edited HSPCs, eight studies from 21 experiments provided the data for RNP, and three studies provided the three experimental data for RNA. Using the fixed effect model, the standardized mean difference yielded a highly significant value for RNP-mediated delivery (95% CI, –0.702 to –0.212; P < 0.001) (Fig. 4c). The analysis revealed altered engraftment of RNP-mediated gene-edited HSPCs, whereas no effect was observed when RNA was used. However, the available data showed a potential funnel plot asymmetry, suggesting a bias in the study (Supplementary Material 7B, jh.elmerpub.com). In total, for the PB engraftment study, 21 studies with 60 experiments provided the data for RNP and eight for RNA from three studies. A fixed effect model was selected to measure the outcome of the standardized mean difference as the data were homogenous and unbiased (Supplementary Material 7C, D, jh.elmerpub.com). The outcome showed the decreased engraftment of RNP and increased engraftment of RNA-mediated gene-edited HSPCs in the spleen (95% CI, –0.388 to –0.096; P = 0.001; 95% CI, 0.046 to 0.649; P = 0.024, respectively) (Fig. 4b, d).

### The implications of mouse strain used in engraftment studies for CRISPR-Cas9-mediated gene-edited HSPCs

**Figure 5:**
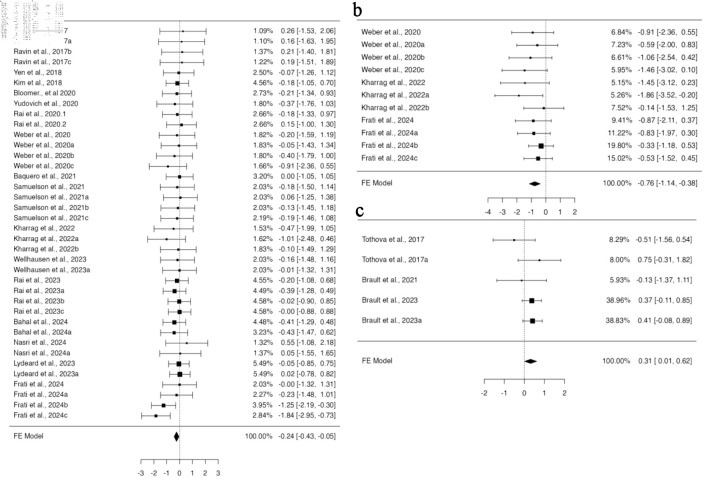
The genetic background of NSGS and NSG Inbred mice influences the engraftment of gene-edited hHSPCs in the spleen, bone marrow, and peripheral blood. (a) The analysis of bone marrow engraftment comprised 38 datasets that used the NSG strain of mice model. The majority of estimates (74%), with observed standardized mean differences ranging from –1.8391 to 0.5537, were negative. (b) Analysis of spleen engraftment was conducted on 11 datasets that used the NSG strain of mice model. All estimations were negative (100%), and the observed standardized mean differences varied from –1.8629 to -0.1399. (c) The analysis comprised five datasets on peripheral blood engraftment using the NSGS mouse strain model. The observed standardized mean differences varied from –0.5092 to 0.7534, with 60% of the estimations being positive. hHSPCs: human hematopoietic stem and progenitor cells.

In the 39 publications included in our study, four distinct mouse strains were used. When employing NBSGW and NSGSGM3 mouse strains, subgroup analysis based on mouse strain using a fixed effect model revealed no difference in the engraftment of edited cells compared to non-edited HSPCs in all four organs investigated. However, when the NSGS mouse model was used for HSPC transplantation, there was a significant increase in the engraftment of HSPCs in the PB (95% CI, 0.011 to 0.615; P = 0.042) (Fig. 5c). The data analyzed using a fixed effect model from five datasets across two studies were homogenous, with no bias inferred from funnel plot asymmetry (Supplementary Material 8C, jh.elmerpub.com). Additional subgroup analysis using the NSG mouse model, based on 38 datasets from 29 studies for BM and 11 studies with 11 datasets for spleen engraftment, indicated a significant reduction in the engraftment of gene-edited cells (95% CI, –0.426 to –0.051; P = 0.013; 95% CI, –1.145 to –0.383; P < 0.001, respectively) (Fig. 5a, b). Neither the rank correlation nor the regression test indicated any funnel plot asymmetry in the data provided for the BM; however, the data for spleen showed some bias (Supplementary Material 8A, B, jh.elmerpub.com).

### Impact of different cell delivery methods for CRISPR-Cas9-mediated gene-edited HSPCs on organ-specific engraftment

**Figure 6:**
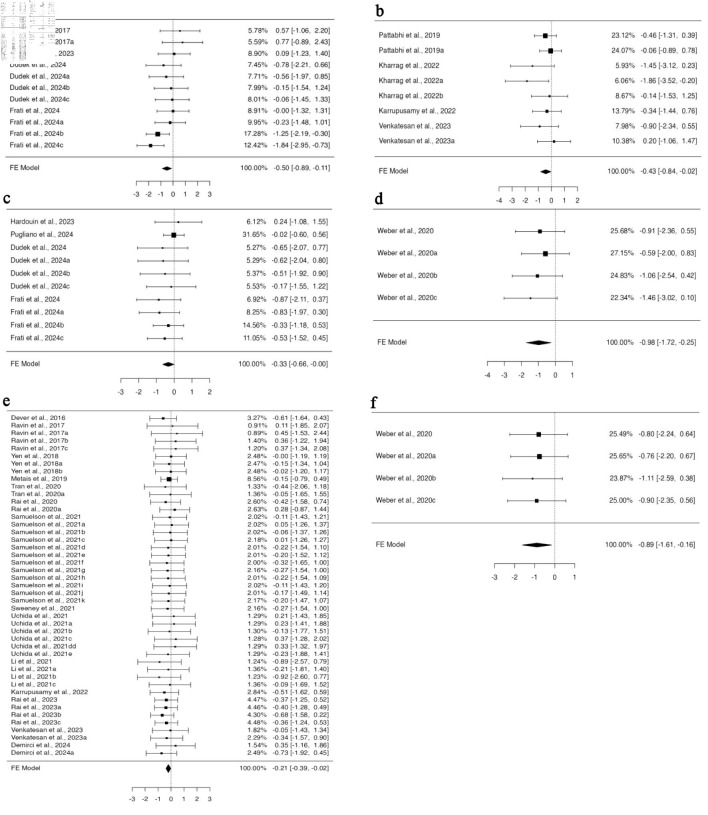
Utilizing the tail vein, intraperitoneal, and retro-orbital routes to deliver cells compromises the engraftment efficiency of gene-edited hHSPCs in the spleen and peripheral blood. The analysis of spleen engraftment comprised (a) eight datasets on spleen engraftment that used the tail vein as the delivery route. The observed standardized mean differences varied from –1.8629 to 0.2046, with 88% of estimates being negative. (b) Ten datasets that used retro-orbital administration. The observed standardized mean differences varied from –0.8680 to 0.2357, with 90% of the estimations being negative. (c) A total of four datasets that used intraperitoneal as the delivery method. All estimations were negative (100%), and the observed standardized mean differences varied from –1.4638 to -0.5878. In order to analyze peripheral blood engraftment (d), 45 datasets that used tail vein injection were considered. A significant portion of estimates (73%), with observed standardized mean differences ranging from –0.9161 to 0.4505, were negative. (e) A total of four datasets with intraperitoneal delivery were included. All estimations were negative (100%), and the observed standardized mean differences varied from –1.1051 to –0.7621. hHSPCs: human hematopoietic stem and progenitor cells.

We categorized the studies into subgroups based on the different routes of cell administration. Five distinct routes of cell administration were identified across all 39 studies. In all, 11 experiments from four studies provided data for BM engraftment through the retro-orbital route of transplantation, whose funnel plot was asymmetrical for the regression test but not for the correlation one (Supplementary Material 9A, jh.elmerpub.com). Fixed model analysis revealed deficient engraftment of gene-edited cells compared to unedited ones (95% CI, –0.894 to –0.109; P = 0.012) (Fig. 6a). For spleen engraftment, eight datasets from four studies utilized tail vein injection, 10 datasets from four studies utilized the retro-orbital route, and four datasets from one study preferred the intraperitoneal route. Data provided in all subgroups were independently analyzed using a fixed effect model. There was no heterogeneity or bias in the data of any subgroup (Supplementary Material 9B–D, jh.elmerpub.com); however, the engraftment potential of gene-edited cells was found to be minimized. The standardized mean difference was statistically significant in the tail vein (95% CI, –0.837 to –0.019; P = 0.04) (Fig. 6b), retro-orbital (95% CI, –0.657 to –0.004; P = 0.047) (Fig. 6c), and intra-peritoneal (95% CI, –1.721 to –0.245; P = 0.009) (Fig. 6d) subgroups.

In total, 14 studies provided 45 datasets on PB engraftment via tail vein transplantation, nine datasets from four studies for intrahepatic, eight datasets from four articles for retro-orbital, four datasets for intraperitoneal, and two datasets for intra-femoral from one study each. The cells transplanted through the tail vein and intraperitoneal route engrafted less efficiently when edited with CRISPR-Cas9 compared to non-edited ones. A fixed effect model was fitted to the analysis, which yielded a statistically significant value for the standardized mean differences (95% CI, –0.394 to –0.020; P = 0.030) for the tail vein (Fig. 6e) and (95% CI, –1.614 to –0.160; P = 0.017) for the intraperitoneal (Fig. 6f) routes. No heterogeneity was found in the data for either subgroup. Neither the rank correlation nor the regression test indicated any funnel plot asymmetry for the data in the intraperitoneal subgroup (P = 0.0833 and P = 0.7220, respectively), while the rank correlation test indicated funnel plot asymmetry (P = 0.0127) for the tail vein subgroup, indicating bias in the data (Supplementary Material 9E, F, jh.elmerpub.com).

### The influence of several tissue-specific HSPCs obtained from various sources on cell engraftment after gene editing

**Figure 7:**
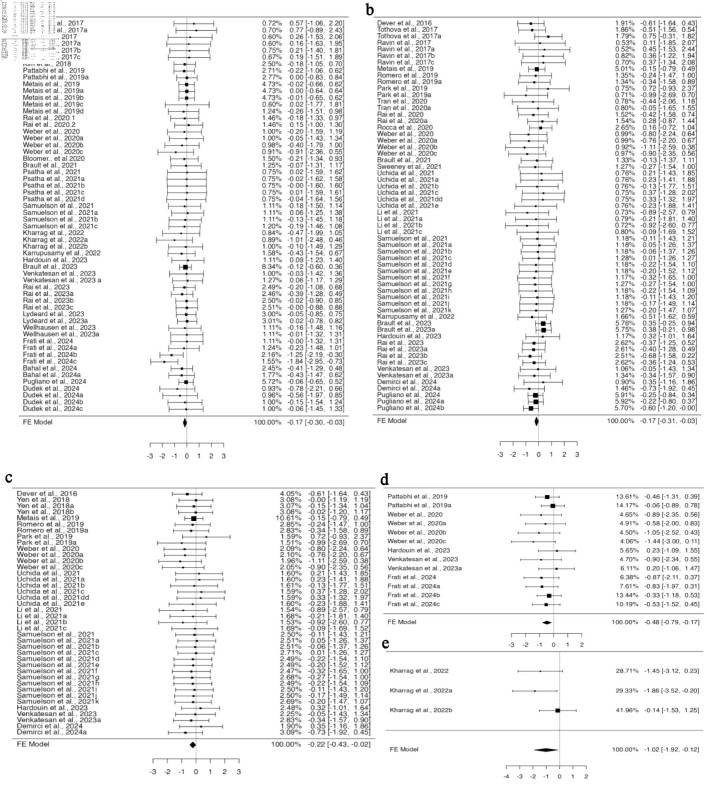
The engraftment capacity of cells is affected by CRISPR-Cas9-based gene editing of peripheral blood-derived HSPCs and hemoglobinopathy-related genes. The investigation of HSPCs obtained from peripheral blood in (a) bone marrow engraftment included 58 datasets in total. With 72% of estimations being negative, the observed standardized mean differences varied from –1.8391 to 0.7740. (b) Peripheral blood engraftment included a total of 60 datasets. The observed standardized mean differences ranged from –1.1051 to 0.7534, with 70% of estimations being negative. To examine the editing effect of hemoglobinopathy-related genes (c) on peripheral blood engraftment, 40 datasets were analyzed. The majority of estimates (78%), with observed standardized mean differences ranging from –1.1051 to 0.7224, were negative. (d) For spleen engraftment, data from 13 datasets were utilized. Most estimates (85%) were negative, and the observed standardized mean differences varied from –1.4431 to 0.2338. CRISPR-Cas9: clustered regularly interspaced short palindromic repeats and CRISPR-associated protein 9; HSPCs: hematopoietic stem and progenitor cells.

There are four sources of HSPCs: BM, PB, UCB, and fetal liver, from which cells are obtained for gene editing and transplantation. After analysis using a fixed effect model, HSPCs from PB showed reduced engraftment in the BM and PB. In BM engraftment studies, 58 investigations from 22 publications used HSPCs from PB, and six datasets each from BM and UCB from three and four publications, respectively. The analysis was carried out using the standardized mean difference as the outcome measure, using a fixed effect model that showed a significant difference in the outcome for HSPCs from PB (95% CI, –0.305 to –0.027; P = 0.019) (Fig. 7a). Of the 25 studies that provided data on PB engraftment, 23 publications with 60 datasets used PB-derived HSPCs, while one each with four datasets used BM- and UCB-derived HSPCs. The estimated average standardized mean difference based on the fixed effects model differed significantly (95% CI, –0.324 to –0.035; P = 0.015), suggesting decreased engraftment of PB-derived gene-edited HSPCs in PB (Fig. 7b). There was no heterogeneity in the data, nor was there any funnel plot asymmetry (Supplementary Material 10A, B, jh.elmerpub.com).

### The impact of disease-specific gene editing using the CRISPR-Cas9 approaches on HSPC engraftment efficiency

A total of 18 gene loci were targeted across the 39 studies included in this analysis, which were associated with five distinct diseases: human immunodeficiency virus (*CCR5*), immune-mediated disease (*CYBB*, *ITGB2*, *FOXP3*, *MAGT1*, *WAS*, *BTK*, *CD40L*), hemoglobinopathies (*HBB*, *HBG*, *BCL11*), leukemia (*TET2*, cohesin, *CD33*, *CD45*), and severe congenital neutropenia (*ELANE*). Transplantation of gene-edited HSPCs in various disease conditions improved the overall outcomes in mouse models. The engraftment of CRISPR-Cas9-mediated genetically modified HSPCs was found to be comparable to that of unedited HSPCs in all disease conditions analyzed in the BM and thymus. In the spleen and PB, except for hemoglobinopathy, gene-edited HSPCs for other targeted disease conditions engrafted similarly to the control. Homogeneous and unbiased data on PB engraftment of HSPCs for CRISPR-Cas9 based modifications of genetic loci involved in hemoglobinopathy were provided by 12 studies with 40 datasets (Supplementary Material 11C, jh.elmerpub.com). The engraftment of gene-edited HSPCs was found to be diminished when analyzed using the fixed effect model (95% CI, –0.432 to –0.016; P = 0.035) (Fig. 7c).

A total of five studies provided 13 datasets for spleen engraftment targeting hemoglobinopathy, which were biased (Supplementary Material 11A, jh.elmerpub.com), and three datasets from one study targeted leukemia genes, which were homogenous and unbiased (Supplementary Material 11B, jh.elmerpub.com). A fixed effect model analysis revealed less engraftment of gene-edited cells targeted for hemoglobinopathy compared to non-edited cells (95% CI, –0.793 to –0.166; P = 0.003) (Fig. 7d) and leukemia genes (95% CI, –1.919 to –0.121; P = 0.026) (Fig. 7e).

## Discussion

Allogeneic HSPC therapy for hereditary blood disorders is limited by donor availability and immunological complications, whereas autologous HSPC gene therapy offers a safer alternative. Recent advances in vector-mediated gene addition and CRISPR-Cas9 gene editing have expanded the therapeutic potential of genetic disorders. Preclinical studies remain crucial for assessing the engraftment efficiency of edited HSPCs in organs such as the BM, spleen, thymus, and PB. Our systematic review and meta-analysis showed that gene-edited cells exhibit lower engraftment efficiency than unmodified cells, except in the thymus. Subgroup analysis revealed that knock-in cells were engrafted similarly to unaltered cells, whereas knockout cells showed reduced engraftment, reflecting the challenges of HDR, which requires the co-delivery of a donor template (ssODN or AAV6). No significant difference in engraftment was observed between the HDR templates, suggesting that the template type may not influence engraftment outcomes. Enhancing HSPC proliferation may partially mitigate reduced engraftment but often compromises stemness, emphasizing the need for improved gene editing techniques (Table 3).

**Table 3:** Practical Recommendations for Optimizing Engraftment

	Gene editing	CRISPR-Cas9 system	Mice strain	Route of transplantation	Source of HSPCs	HDR template
Bone marrow	Knock-in	RNA	NSGS	Tail vein	BM	AAV6
			NBSGW	Intrahepatic	UCB	ssODN
			NSGSGM3	Intraperitoneal		
Spleen	Knock-in	RNP	NBSGW	Intrahepatic?	Unknown	AAV6
			NSGSGM3			
Thymus	Knock-in	RNA	NSG	Tail vein	Unknown	AAV6
	Knockout	RNP	NSGS	Intrahepatic		
				Intraperitoneal		
Peripheral blood	Knock-in	Plasmid?	NBSGW	Intrahepatic	BM	AAV6
			NSGSGM3	Retro-orbital	UCB	ssODN

CRISPR-Cas9: clustered regularly interspaced short palindromic repeats and CRISPR-associated protein 9; HSPCs: hematopoietic stem and progenitor cells; HDR: homology-directed repair; RNP: ribonucleoprotein complex; BM: bone marrow; UCB: umbilical cord blood; AAV6: adeno-associated virus type 6; ssODN: single-strand oligonucleotide.

CRISPR-Cas9 enables precise and regulated gene correction, avoiding the genotoxic risks associated with semi-random viral vector integration. Nevertheless, the success of this platform is contingent upon the meticulous selection of reagents to achieve a balance between efficacy, specificity, and safety while minimizing cytotoxicity, especially in self-renewing HSPCs. Our subgroup analysis revealed that the CRISPR-Cas9 delivery mode significantly influenced engraftment outcomes. Specifically, RNP-based delivery reduced engraftment in both the BM and spleen, whereas both RNP and RNA delivery decreased PB engraftment. Although plasmid-based CRISPR-Cas9 systems are simpler and prevent multi-component transfection, and Cas9 mRNA systems exhibit reduced stability, the limited data available preclude robust statistical conclusions. Crucially, further investigation is required to assess the genome integrity of edited HSPCs, as emerging evidence indicates the potential risks of chromosomal arm loss, translocations, deletions, and chromothripsis following nuclease treatment [58–60].

Engraftment outcomes are influenced by the HSPC source. Each source BM, PB, or cord blood has distinct advantages regarding engraftment kinetics, immune recovery, and GVHD risk. Preclinical studies have relied on animal models to explore HSPC transplantation and gene editing. Repeated HSPC transplantation leads to exhaustion, although primary transplants achieve robust engraftment across organs [61]. Our findings showed that the administration route and animal strain affected engraftment potential. Spleen engraftment diminished when cells were delivered via the tail vein, intraperitoneal, or retro-orbital routes, whereas PB engraftment was compromised following tail vein or intraperitoneal injection. The spleen, a potential extramedullary hematopoietic site, contributes to early cytokine-driven engraftment reactions. Splenectomy facilitates early engraftment, whereas splenomegaly delays it, possibly explaining the diminished splenic engraftment [62].

Engraftment differences across mouse strains reflect immune modulation. NSG and NSGS mice, which lack T, B, and natural killer (NK) cells, showed lower engraftment of gene-edited HSPCs than NBSGW and NSG-SGM3 strains, which possess modifications facilitating enhanced human HSPC engraftment [63]. While pooled analyses indicated reduced persistence of gene-edited HSPCs in the BM, disease-specific subgroups showed no consistent impact on transplantation efficiency. In HIV, immune-mediated disorders, hemoglobinopathies, leukemia, and severe congenital neutropenia models, CRISPR-Cas9–corrected HSPCs maintained robust engraftment. Gene-edited HSPCs restored immune function and conferred HIV-1 resistance; corrected hemoglobinopathy models showed reduced HbS levels, mimicking asymptomatic phenotypes; and edited HSPCs improved outcomes in immune-mediated disease and neutropenia models. In chimeric antigen receptor T-cells (CAR-T) modifications, HSPC gene editing reduces tumor burden while preserving stem cell function. CRISPR-Cas9–based HSPC gene therapy shows promise but faces challenges in optimizing delivery, preserving stemness, and ensuring genomic stability. Understanding the effects of editing on engraftment will be crucial for translating preclinical success into clinical therapies.

### Limitations

The nonhuman primate (NHP) autologous transplantation model is a valuable tool for assessing the long-term durability of gene-modified cells; however, it was excluded because it does not use human hematopoietic cells. Our analysis is subject to several limitations, including the lack of reported blinding and randomization, unaccounted *ex vivo* HSPC culture conditions and cell doses, and uneven study weighting resulting from variance differences. These issues highlight the necessity of standardized protocols in transplantation, cell culture, and gene editing, as well as preregistered study designs. The standardization of these procedures would facilitate more comprehensive information synthesis initiatives, which should hasten the transition to clinical trials.

## Supplementary Material

Suppl 1 Assessment of engraftment percentage in bone marrow for CRISPR-Cas9-edited HSPCs.

Suppl 2 Funnel plot for pooled analysis.

Suppl 3 Comprehensive evaluation of CRISPR-Cas9 gene-edited HSPC engraftment in spleen.

Suppl 4 Evaluation of the percentage of CRISPR-Cas9 gene-edited HSPCs that engraft in peripheral blood.

Suppl 5 Comprehensive measurement of engraftment for CRISPR-Cas9 gene-edited HSPCs in thymus.

Suppl 6 Funnel plot of knockout subgroup analysis.

Suppl 7 Funnel plot of subgroup for CRISPR system analysis.

Suppl 8 Funnel plot of mice strain subgroup analysis.

Suppl 9 Funnel plot for subgroup route of delivery analysis.

Suppl 10 Funnel plot for the subgroup analysis of peripheral blood as source of HSPC analysis.

Suppl 11 Funnel plot for subgroup analysis of disease-targeted and HDR analyses.

## Data Availability

The authors declare that data supporting the findings of this study are available within the article.
