# Supplementary material for: Engraftment Outcome of CRISPR/Cas9-Edited Hematopoietic Stem Cells for Genetic Diseases: A Systematic Review and Meta-Analysis of Preclinical Evidence
Source: J Hematol. 2026 Apr 6;15(2):108–28. doi: 10.14740/jh2190 (PMC13071946; doi:10.14740/jh2190)
Supplement: Suppl 1 — Assessment of engraftment percentage in bone marrow for CRISPR-Cas9-edited HSPCs. [file jh-15-02-108-s001.docx]

**Suppl 1. Assessment of engraftment percentage in bone marrow for CRISPR-Cas9 edited HSPCs.**

| Author and Year | Gene edited | | | Unedited | | |
| --- | --- | --- | --- | --- | --- | --- |
|  | Mean | SD | N | Mean | SD | N |
| Tothova et al., 2017 | 44.158 | 44.990 | 3 | 19.110 | 21.190 | 3 |
| Tothova et al., 2017a | 69.483 | 70.315 | 3 | 19.110 | 21.190 | 3 |
| Ravin et al., 2017 | 68.509 | 75.834 | 3 | 44.120 | 44.542 | 2 |
| Ravin et al., 2017a | 58.392 | 74.167 | 3 | 44.120 | 44.542 | 2 |
| Ravin et al., 2017b | 62.077 | 80.528 | 6 | 44.120 | 44.542 | 2 |
| Ravin et al., 2017c | 60.551 | 76.890 | 4 | 44.120 | 44.542 | 2 |
| Kim et al., 2018 | 36.786 | 59.619 | 10 | 49.789 | 80.867 | 10 |
| Yen et al., 2018 | 30.051 | 37.580 | 6 | 33.037 | 41.920 | 5 |
| Pattabhi et al., 2019 | 64.071 | 81.797 | 17 | 83.393 | 92.256 | 8 |
| Pattabhi et al., 2019a | 83.861 | 96.385 | 18 | 83.393 | 92.256 | 8 |
| Borot et al., 2019 | 20.540 | 11.039 | 8 | 30.775 | 8.603 | 6 |
| Borot et al., 2019a | 62.521 | 23.712 | 8 | 69.258 | 15.755 | 6 |
| Metais et al., 2019 | 89.375 | 97.917 | 23 | 91.458 | 96.042 | 16 |
| Metais et al., 2019a | 78.436 | 87.868 | 23 | 78.339 | 89.340 | 16 |
| Metais et al., 2019b | 70.576 | 77.452 | 23 | 71.658 | 78.927 | 16 |
| Metais et al., 2019c | 92.498 | 94.974 | 3 | 89.499 | 91.785 | 2 |
| Metais et al., 2019d | 55.261 | 69.546 | 5 | 78.165 | 86.737 | 5 |
| Yudovich et al., 2020 | 38.374 | 51.296 | 4 | 64.703 | 71.621 | 4 |
| Rai et al., 2020 | 36.594 | 54.277 | 7 | 46.972 | 54.244 | 5 |
| Rai et al., 2020 | 57.305 | 68.286 | 7 | 46.972 | 54.244 | 5 |
| Goodwin et al., 2020 | 16.459 | 17.847 | 2 | 0.397 | 1.190 | 2 |
| Goodwin et al., 2020a | 16.856 | 27.365 | 2 | 0.397 | 1.785 | 2 |
| Goodwin et al., 2020b | 29.943 | 67.819 | 3 | 0.397 | 1.586 | 3 |
| Weber et al., 2020 | 39.489 | 19.618 | 4 | 45.223 | 29.428 | 4 |
| Weber et al., 2020a | 43.836 | 19.074 | 4 | 45.223 | 29.428 | 4 |
| Weber et al., 2020b | 34.560 | 15.259 | 4 | 45.223 | 29.428 | 4 |
| Weber et al., 2020c | 19.562 | 18.528 | 4 | 45.223 | 29.428 | 4 |
| Brault et al., 2021 | 69.158 | 71.881 | 3 | 74.644 | 77.368 | 15 |
| Bloomer., et al 2020 | 1.295 | 3.172 | 6 | 2.330 | 5.707 | 6 |
| Psatha et al., 2021 | 85.511 | 148.110 | 3 | 83.505 | 8.660 | 3 |
| Psatha et al., 2021a | 81.268 | 140.760 | 3 | 83.505 | 8.660 | 3 |
| Psatha et al., 2021b | 83.275 | 144.236 | 3 | 83.505 | 8.660 | 3 |
| Psatha et al., 2021c | 85.282 | 147.712 | 3 | 83.505 | 8.660 | 3 |
| Psatha et al., 2021d | 78.288 | 135.599 | 3 | 83.505 | 8.660 | 3 |
| Baquero et al., 2021 | 87.242 | 93.048 | 7 | 87.216 | 95.442 | 7 |
| Samuelson et al., 2021 | 40.771 | 61.067 | 5 | 55.167 | 81.383 | 4 |
| Samuelson et al., 2021a | 61.260 | 88.321 | 5 | 55.167 | 81.383 | 4 |
| Samuelson et al., 2021b | 43.904 | 71.600 | 5 | 55.167 | 81.383 | 4 |
| Samuelson et al., 2021c | 40.925 | 57.415 | 6 | 55.167 | 81.383 | 4 |
| Kharrag et al., 2022 | 82.119 | 9.797 | 3 | 86.315 | 5.474 | 4 |
| Kharrag et al., 2022a | 86.602 | 9.269 | 4 | 94.996 | 4.278 | 4 |
| Kharrag et al., 2022b | 94.607 | 2.139 | 4 | 94.996 | 4.278 | 4 |
| Karrupusamy et al., 2022 | 70.732 | 19.338 | 9 | 79.443 | 17.920 | 5 |
| Hardouin et al., 2023 | 97.315 | 8.087 | 5 | 96.658 | 3.945 | 4 |
| Brault et al., 2023 | 21.705 | 32.875 | 50 | 25.679 | 35.793 | 25 |
| Lydeard et al., 2023 | 73.414 | 89.333 | 12 | 78.317 | 89.760 | 12 |
| Lydeard et al., 2023a | 83.038 | 92.989 | 12 | 80.978 | 95.405 | 12 |
| Venkatesan et al., 2023 | 87.271 | 213.769 | 6 | 94.070 | 162.934 | 3 |
| Venkatesan et al., 2023 a | 75.394 | 199.473 | 7 | 64.162 | 128.323 | 4 |
| Wellhausen et al., 2023 | 57.086 | 70.659 | 5 | 70.459 | 79.641 | 4 |
| Wellhausen et al., 2023a | 69.860 | 90.020 | 5 | 70.459 | 79.641 | 4 |
| Rai et al., 2023 | 20.844 | 31.010 | 10 | 28.074 | 37.073 | 10 |
| Rai et al., 2023a | 14.026 | 31.193 | 10 | 28.074 | 37.073 | 10 |
| Rai et al., 2023b | 27.042 | 43.042 | 10 | 28.074 | 37.073 | 10 |
| Rai et al., 2023c | 28.058 | 40.141 | 10 | 28.074 | 37.073 | 10 |
| Bahal et al., 2024 | 14.680 | 21.610 | 10 | 26.149 | 31.298 | 10 |
| Bahal et al., 2024a | 5.222 | 8.984 | 9 | 11.642 | 19.860 | 6 |
| Nasri et al., 2024 | 38.871 | 44.134 | 3 | 13.045 | 28.674 | 3 |
| Nasri et al., 2024a | 68.080 | 78.130 | 3 | 62.743 | 79.828 | 3 |
| Pugliano et al., 2024 | 65.910 | 76.820 | 25 | 71.083 | 80.174 | 21 |
| Dudek et al., 2024 | 1.384 | 1.509 | 4 | 6.691 | 8.262 | 4 |
| Dudek et al., 2024a | 2.538 | 3.917 | 4 | 6.691 | 8.262 | 4 |
| Dudek et al., 2024b | 5.334 | 7.184 | 4 | 6.691 | 8.262 | 4 |
| Dudek et al., 2024c | 5.958 | 12.539 | 4 | 6.691 | 8.262 | 4 |
| Frati et al., 2024 | 86.434 | 5.833 | 4 | 86.449 | 8.851 | 5 |
| Frati et al., 2024a | 84.336 | 7.454 | 5 | 86.449 | 8.851 | 5 |
| Frati et al., 2024b | 44.660 | 14.141 | 8 | 66.341 | 17.929 | 14 |
| Frati et al., 2024c | 35.479 | 9.696 | 6 | 66.341 | 17.929 | 14 |
