# Supplementary material for: Engraftment Outcome of CRISPR/Cas9-Edited Hematopoietic Stem Cells for Genetic Diseases: A Systematic Review and Meta-Analysis of Preclinical Evidence
Source: J Hematol. 2026 Apr 6;15(2):108–28. doi: 10.14740/jh2190 (PMC13071946; doi:10.14740/jh2190)
Supplement: Suppl 2 — Funnel plot for pooled analysis. [file jh-15-02-108-s002.docx]

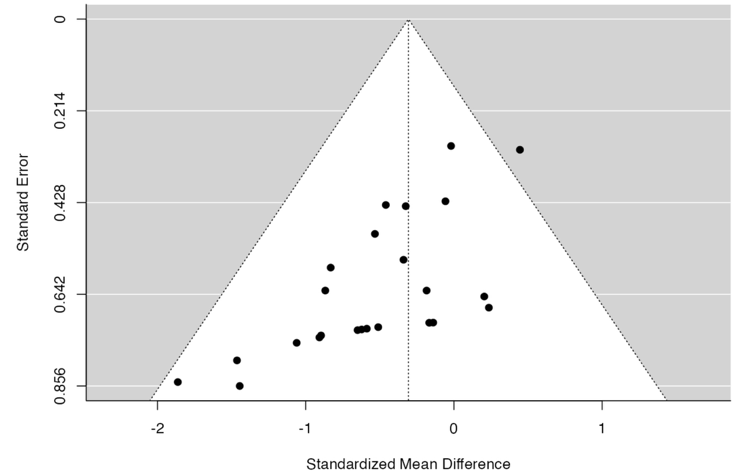

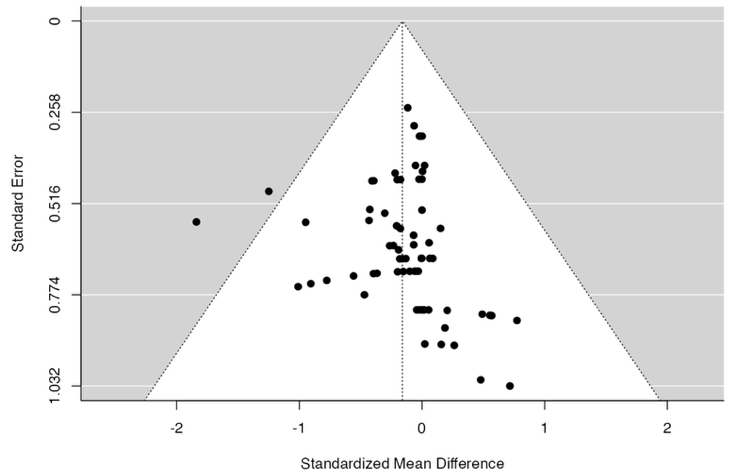
 A BM B Spleen


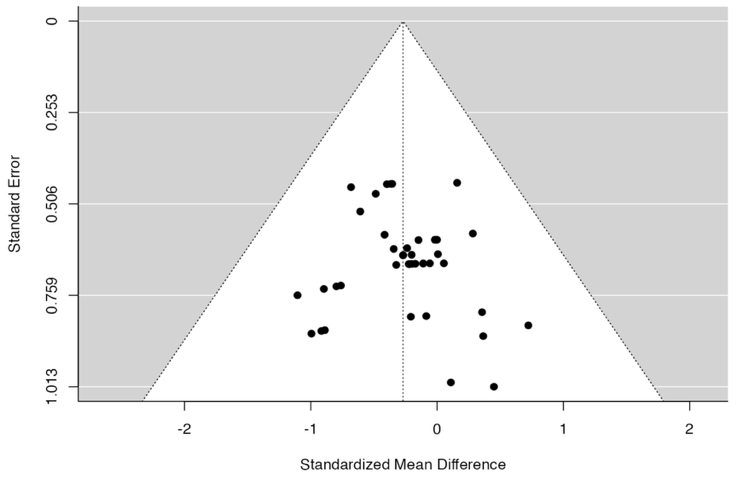
C PB

**Suppl 2.** Funnel plot for pooled analysis. (A) For bone marrow, the rank correlation and the regression test indicated potential funnel plot symmetry (p=0.1373 and p = 0.7823, respectively) (B) For spleen engraftment the rank correlation and the regression test indicated potential funnel plot asymmetry (p < 0.0001 and p = 0.0016, respectively) (C) For peripheral blood the data provided are homogenous without any bias (Correlation p = 0.9023 and Regression p = 0.7030).
