# Supplementary material for: Engraftment Outcome of CRISPR/Cas9-Edited Hematopoietic Stem Cells for Genetic Diseases: A Systematic Review and Meta-Analysis of Preclinical Evidence
Source: J Hematol. 2026 Apr 6;15(2):108–28. doi: 10.14740/jh2190 (PMC13071946; doi:10.14740/jh2190)
Supplement: Suppl 3 — Comprehensive evaluation of CRISPR-Cas9 gene-edited HSPC engraftment in spleen. [file jh-15-02-108-s003.docx]

**Suppl 3. Comprehensive evaluation of CRISPR-cas9 gene-edited HSPC engraftment in spleen.**

| Author and Year | Gene edited | | | Unedited | | |
| --- | --- | --- | --- | --- | --- | --- |
|  | Mean | SD | N | Mean | SD | N |
| Pattabhi et al., 2019 | 11.077 | 16.665 | 17 | 22.305 | 34.636 | 8 |
| Pattabhi et al., 2019a | 20.461 | 29.709 | 18 | 22.305 | 34.636 | 8 |
| Weber et al., 2020 | 24.706 | 8.720 | 4 | 48.151 | 30.518 | 4 |
| Weber et al., 2020a | 32.051 | 14.168 | 4 | 48.151 | 30.518 | 4 |
| Weber et al., 2020b | 20.867 | 8.175 | 4 | 48.151 | 30.518 | 4 |
| Weber et al., 2020c | 9.955 | 9.809 | 4 | 48.151 | 30.518 | 4 |
| Brault et al., 2021 | 47.373 | 52.822 | 3 | 59.475 | 63.755 | 15 |
| Kharrag et al., 2022 | 71.536 | 5.689 | 3 | 84.308 | 8.393 | 4 |
| Kharrag et al., 2022a | 63.907 | 14.259 | 4 | 90.303 | 9.981 | 4 |
| Kharrag et al., 2022b | 88.666 | 10.338 | 4 | 90.303 | 9.981 | 4 |
| Karrupusamy et al., 2022 | 41.494 | 36.126 | 9 | 54.320 | 33.561 | 5 |
| Hardouin et al., 2023 | 79.562 | 11.762 | 5 | 75.945 | 15.781 | 4 |
| Brault et al., 2023 | 8.869 | 14.595 | 50 | 2.937 | 4.834 | 14 |
| Venkatesan et al., 2023 | 70.184 | 11.891 | 6 | 81.118 | 7.567 | 3 |
| Venkatesan et al., 2023a | 48.308 | 16.696 | 6 | 43.951 | 22.815 | 4 |
| Pugliano et al., 2024 | 11.321 | 19.245 | 25 | 11.698 | 19.245 | 21 |
| Dudek et al., 2024 | 0.281 | 0.303 | 4 | 6.987 | 12.671 | 4 |
| Dudek et al., 2024a | 0.555 | 0.712 | 4 | 6.987 | 12.671 | 4 |
| Dudek et al., 2024b | 1.652 | 1.990 | 4 | 6.987 | 12.671 | 4 |
| Dudek et al., 2024c | 4.562 | 12.614 | 4 | 6.987 | 12.671 | 4 |
| Frati et al., 2024 | 78.463 | 5.590 | 5 | 86.186 | 9.696 | 6 |
| Frati et al., 2024a | 77.407 | 9.922 | 7 | 86.186 | 9.696 | 6 |
| Frati et al., 2024b | 36.065 | 8.125 | 9 | 38.996 | 9.014 | 13 |
| Frati et al., 2024c | 34.175 | 7.655 | 6 | 38.996 | 9.014 | 13 |
