# Supplementary material for: Engraftment Outcome of CRISPR/Cas9-Edited Hematopoietic Stem Cells for Genetic Diseases: A Systematic Review and Meta-Analysis of Preclinical Evidence
Source: J Hematol. 2026 Apr 6;15(2):108–28. doi: 10.14740/jh2190 (PMC13071946; doi:10.14740/jh2190)
Supplement: Suppl 4 — Evaluation of the percentage of CRISPR-Cas9 gene-edited HSPCs that engraft in peripheral blood. [file jh-15-02-108-s004.docx]

**Suppl 4. Evaluation of the percentage of CRISPR-cas9 gene-edited HSPCs that engraft in peripheral blood.**

| Author and Year | Gene edited | | | Unedited | | |
| --- | --- | --- | --- | --- | --- | --- |
|  | Mean | SD | N | Mean | SD | N |
| Dever et al., 2016 | 21.700 | 2.600 | 6 | 29.300 | 14.600 | 10 |
| Lei Xu et al., 2017 | 9.564 | 4.888 | 9 | 12.871 | 7.753 | 9 |
| Tothova et al., 2017 | 6.597 | 7.886 | 9 | 18.066 | 32.690 | 6 |
| Tothova et al., 2017a | 60.217 | 61.938 | 9 | 18.066 | 32.690 | 6 |
| Ravin et al., 2017 | 12.500 | 19.435 | 2 | 9.171 | 14.705 | 2 |
| Ravin et al., 2017a | 33.204 | 39.942 | 2 | 9.171 | 14.705 | 2 |
| Ravin et al., 2017b | 20.533 | 30.018 | 7 | 9.171 | 14.705 | 2 |
| Ravin et al., 2017c | 18.564 | 22.103 | 4 | 9.171 | 14.705 | 2 |
| Yen et al., 2018 | 4.173 | 5.847 | 6 | 4.177 | 5.183 | 5 |
| Yen et al., 2018a | 1.980 | 3.306 | 6 | 2.527 | 3.493 | 5 |
| Yen et al., 2018b | 3.385 | 5.213 | 6 | 3.481 | 4.684 | 5 |
| Metais et al., 2019 | 11.875 | 18.750 | 23 | 15.000 | 22.708 | 16 |
| Romero et al., 2019 | 2.251 | 7.088 | 7 | 5.635 | 20.233 | 4 |
| Romero et al., 2019a | 1.207 | 1.910 | 7 | 5.635 | 20.233 | 4 |
| Park et al., 2019 | 42.000 | 6.000 | 3 | 37.000 | 5.000 | 3 |
| Park et al., 2019a | 7.500 | 9.000 | 3 | 16.800 | 5.500 | 3 |
| Tran et al., 2020 | 0.370 | 0.571 | 3 | 1.448 | 2.700 | 3 |
| Tran et al., 2020a | 0.249 | 0.338 | 3 | 0.270 | 0.384 | 3 |
| Rai et al., 2020 | 15.837 | 24.240 | 7 | 29.751 | 38.784 | 5 |
| Rai et al., 2020a | 47.511 | 67.678 | 7 | 29.751 | 38.784 | 5 |
| Rocca et al., 2020 | 27.299 | 25.235 | 10 | 23.258 | 23.556 | 10 |
| Goodwin et al., 2020 | 22.379 | 33.766 | 5 | 18.667 | 25.103 | 6 |
| Goodwin et al., 2020a | 43.216 | 76.137 | 5 | 22.379 | 33.766 | 5 |
| Goodwin et al., 2020b | 63.805 | 82.369 | 5 | 65.785 | 72.220 | 6 |
| Goodwin et al., 2020c | 91.819 | 97.512 | 5 | 82.660 | 96.522 | 6 |
| Weber et al., 2020 | 13.123 | 5.450 | 4 | 24.307 | 16.349 | 4 |
| Weber et al., 2020a | 13.383 | 6.539 | 4 | 24.307 | 16.349 | 4 |
| Weber et al., 2020b | 8.466 | 6.539 | 4 | 24.307 | 16.349 | 4 |
| Weber et al., 2020c | 11.452 | 6.539 | 4 | 24.307 | 16.349 | 4 |
| Brault et al., 2021 | 52.235 | 59.239 | 3 | 62.001 | 70.561 | 15 |
| Sweeney et al., 2021 | 15.716 | 21.896 | 6 | 22.965 | 28.058 | 4 |
| Uchida et al., 2021 | 12.761 | 28.535 | 5 | 6.269 | 8.865 | 2 |
| Uchida et al., 2021a | 14.328 | 32.039 | 5 | 6.269 | 8.865 | 2 |
| Uchida et al., 2021b | 4.701 | 10.513 | 5 | 6.269 | 8.865 | 2 |
| Uchida et al., 2021c | 25.075 | 56.069 | 5 | 2.910 | 4.116 | 2 |
| Uchida et al., 2021dd | 12.985 | 29.036 | 5 | 2.910 | 4.116 | 2 |
| Uchida et al., 2021e | 1.791 | 4.005 | 5 | 2.910 | 4.116 | 2 |
| Li et al., 2021 | 1.547 | 1.955 | 3 | 21.187 | 24.861 | 3 |
| Li et al., 2021a | 15.367 | 19.448 | 3 | 21.187 | 24.861 | 3 |
| Li et al., 2021b | 1.547 | 1.955 | 3 | 17.456 | 19.497 | 3 |
| Li et al., 2021c | 15.367 | 19.448 | 3 | 17.456 | 19.497 | 3 |
| Samuelson et al., 2021 | 13.670 | 17.836 | 5 | 16.355 | 26.077 | 4 |
| Samuelson et al., 2021a | 18.114 | 31.077 | 5 | 16.355 | 26.077 | 4 |
| Samuelson et al., 2021b | 14.966 | 17.374 | 5 | 16.355 | 26.077 | 4 |
| Samuelson et al., 2021c | 16.540 | 25.059 | 6 | 16.355 | 26.077 | 4 |
| Samuelson et al., 2021d | 7.718 | 12.903 | 5 | 11.515 | 18.459 | 4 |
| Samuelson et al., 2021e | 8.089 | 12.163 | 5 | 11.515 | 18.459 | 4 |
| Samuelson et al., 2021f | 6.515 | 8.644 | 5 | 11.515 | 18.459 | 4 |
| Samuelson et al., 2021g | 7.255 | 10.959 | 6 | 11.515 | 18.459 | 4 |
| Samuelson et al., 2021h | 6.304 | 10.008 | 5 | 9.545 | 15.841 | 4 |
| Samuelson et al., 2021i | 7.786 | 12.600 | 5 | 9.545 | 15.841 | 4 |
| Samuelson et al., 2021j | 6.860 | 12.138 | 5 | 9.545 | 15.841 | 4 |
| Samuelson et al., 2021k | 6.304 | 13.619 | 6 | 9.545 | 15.841 | 4 |
| Karrupusamy et al., 2022 | 18.915 | 15.968 | 9 | 27.596 | 15.415 | 5 |
| Brault et al., 2023 | 7.126 | 13.249 | 50 | 2.844 | 6.353 | 25 |
| Brault et al., 2023a | 17.860 | 33.205 | 50 | 6.068 | 14.862 | 25 |
| Hardouin et al., 2023 | 72.658 | 10.292 | 5 | 68.055 | 15.781 | 4 |
| Rai et al., 2023 | 3.505 | 5.820 | 10 | 6.588 | 9.809 | 10 |
| Rai et al., 2023a | 3.162 | 6.333 | 10 | 6.588 | 9.809 | 10 |
| Rai et al., 2023b | 1.460 | 2.768 | 10 | 6.588 | 9.809 | 10 |
| Rai et al., 2023c | 3.533 | 6.251 | 10 | 6.588 | 9.809 | 10 |
| Venkatesan et al., 2023 | 39.806 | 97.504 | 6 | 44.175 | 25.224 | 3 |
| Venkatesan et al., 2023a | 8.738 | 5.137 | 7 | 11.650 | 11.650 | 4 |
| Demirci et al., 2024 | 9.322 | 16.342 | 3 | 4.121 | 8.859 | 4 |
| Demirci et al., 2024a | 0.987 | 2.177 | 7 | 7.999 | 13.669 | 5 |
| Pugliano et al., 2024 | 10.491 | 8.423 | 24 | 12.842 | 10.130 | 21 |
| Pugliano et al., 2024a | 10.526 | 8.938 | 24 | 13.123 | 14.310 | 21 |
| Pugliano et al., 2024b | 8.982 | 6.360 | 24 | 15.333 | 13.667 | 21 |
