# Supplementary material for: Engraftment Outcome of CRISPR/Cas9-Edited Hematopoietic Stem Cells for Genetic Diseases: A Systematic Review and Meta-Analysis of Preclinical Evidence
Source: J Hematol. 2026 Apr 6;15(2):108–28. doi: 10.14740/jh2190 (PMC13071946; doi:10.14740/jh2190)
Supplement: Suppl 5 — Comprehensive measurement of engraftment for CRISPR-Cas9 gene-edited HSPCs in thymus. [file jh-15-02-108-s005.docx]

**Suppl 5. Comprehensive measurement of engraftment for CRISPR-cas9 gene-edited HSPCs in thymus.**

| **Author and Year** | Gene Edited | | | Unedited | | |
| --- | --- | --- | --- | --- | --- | --- |
|  | Mean | SD | N | Mean | SD | N |
| Weber et al., 2020 | 97.660 | 195.321 | 4 | 98.218 | 2.725 | 4 |
| Weber et al., 2020a | 97.649 | 195.299 | 4 | 98.218 | 2.725 | 4 |
| Weber et al., 2020b | 98.180 | 196.360 | 4 | 98.218 | 2.725 | 4 |
| Weber et al., 2020c | 97.351 | 194.702 | 4 | 98.218 | 2.725 | 4 |
| Brault et al., 2021 | 26.170 | 30.840 | 3 | 63.563 | 74.068 | 15 |
| Brault et al., 2021a | 57.649 | 64.653 | 12 | 63.563 | 74.068 | 15 |
| Brault et al., 2021b | 58.389 | 65.782 | 24 | 63.563 | 74.068 | 15 |
| Kharrag et al., 2022 | 88.322 | 12.641 | 3 | 95.803 | 6.934 | 4 |
| Kharrag et al., 2022a | 66.165 | 8.555 | 4 | 79.192 | 13.545 | 4 |
| Kharrag et al., 2022b | 91.101 | 7.129 | 4 | 79.192 | 13.545 | 4 |
| Brault et al., 2023 | 13.305 | 31.378 | 50 | 0.523 | 1.975 | 25 |
