# Supplementary material for: Engraftment Outcome of CRISPR/Cas9-Edited Hematopoietic Stem Cells for Genetic Diseases: A Systematic Review and Meta-Analysis of Preclinical Evidence
Source: J Hematol. 2026 Apr 6;15(2):108–28. doi: 10.14740/jh2190 (PMC13071946; doi:10.14740/jh2190)
Supplement: Suppl 6 — Funnel plot of knockout subgroup analysis. [file jh-15-02-108-s006.docx]

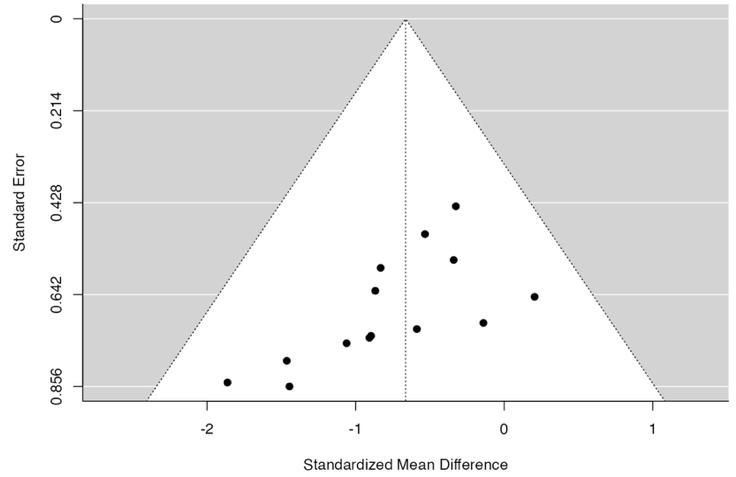

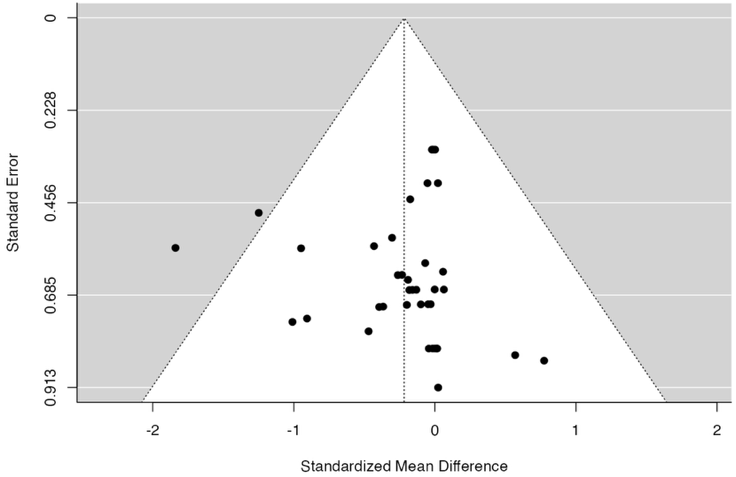
A BM B Spleen


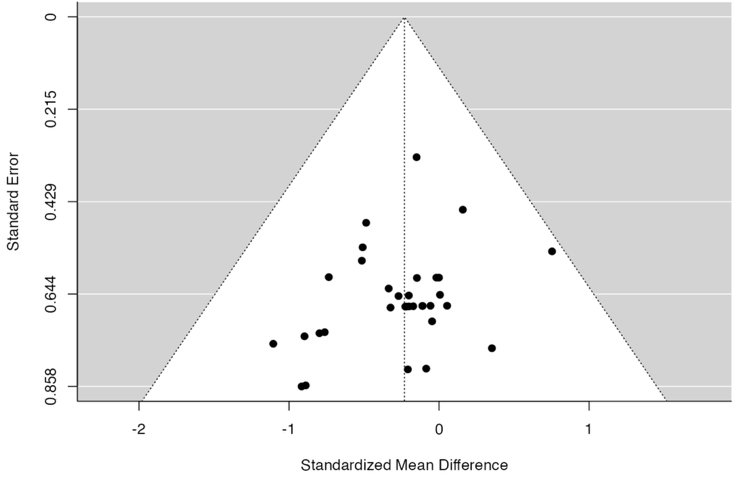


C PB

**Suppl 6.** Funnel plot of knockout subgroup analysis. (A) For bone marrow, the rank correlation and the regression test indicated potential funnel plot symmetry (p=0.920 and p = 0.758, respectively) (B) For spleen engraftment the rank correlation and the regression test indicated potential funnel plot asymmetry (p < 0.001 and p = 0.078, respectively) (C) For peripheral blood the data provided the value for bias as (Correlation p = 0.026 and Regression p = 0.330).
