# Supplementary material for: Engraftment Outcome of CRISPR/Cas9-Edited Hematopoietic Stem Cells for Genetic Diseases: A Systematic Review and Meta-Analysis of Preclinical Evidence
Source: J Hematol. 2026 Apr 6;15(2):108–28. doi: 10.14740/jh2190 (PMC13071946; doi:10.14740/jh2190)
Supplement: Suppl 7 — Funnel plot of subgroup for CRISPR system analysis. [file jh-15-02-108-s007.docx]

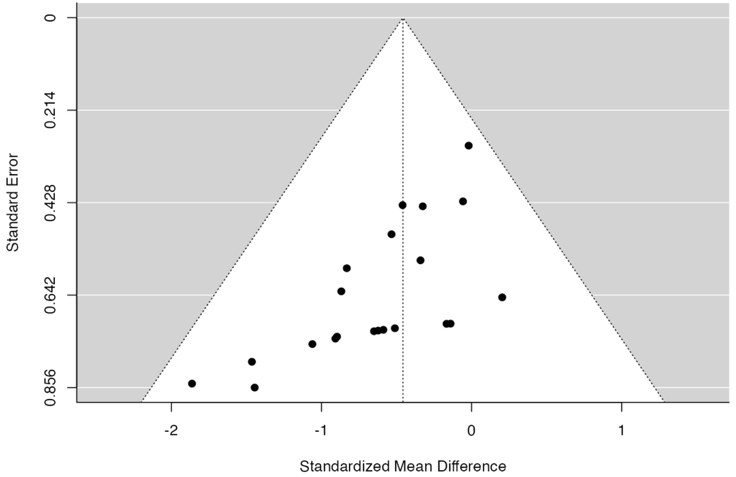

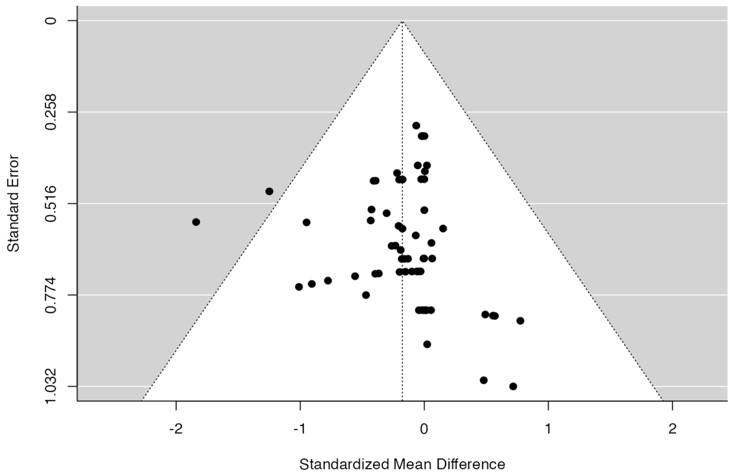
A BM B Spleen


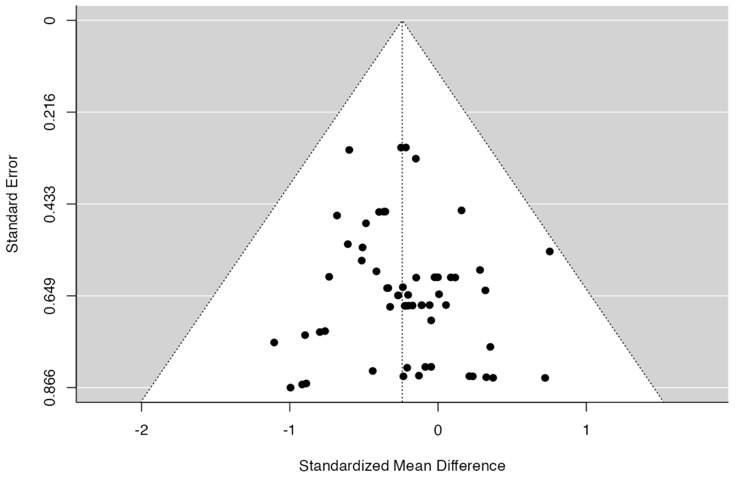

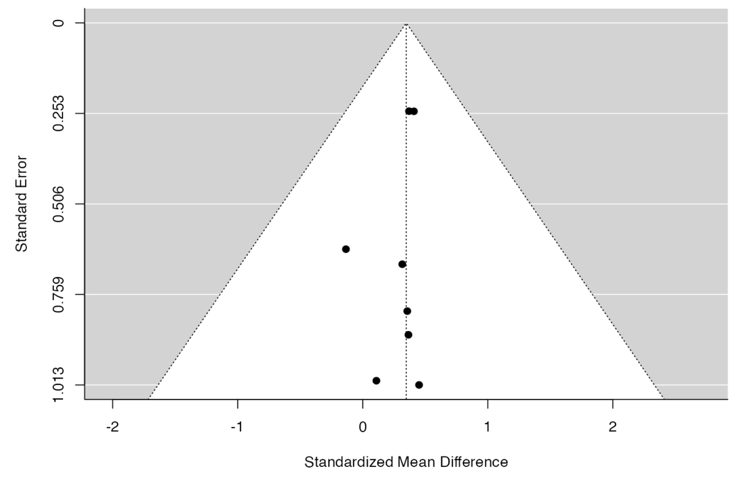
C PB RNP D PB RNA

**Suppl 7.** Funnel plot of subgroup for CRISPR system analysis. (A) For bone marrow, the rank correlation and the regression test indicated potential funnel plot symmetry (p=0.590 and p = 0.922, respectively) (B) For spleen engraftment the rank correlation and the regression test indicated potential funnel plot asymmetry (p < 0.001 and p = 0.012, respectively) (C) For peripheral blood the data for RNP provided the value for unbias as (Correlation p = 0.634 and Regression p = 0.548) (D) For PB engraftment data using RNA as CRISPR system neither the rank correlation nor the regression test indicated any funnel plot asymmetry (p = 0.9049 and p = 0.7042, respectively).
