# Supplementary material for: Engraftment Outcome of CRISPR/Cas9-Edited Hematopoietic Stem Cells for Genetic Diseases: A Systematic Review and Meta-Analysis of Preclinical Evidence
Source: J Hematol. 2026 Apr 6;15(2):108–28. doi: 10.14740/jh2190 (PMC13071946; doi:10.14740/jh2190)
Supplement: Suppl 8 — Funnel plot of mice strain subgroup analysis. [file jh-15-02-108-s008.docx]

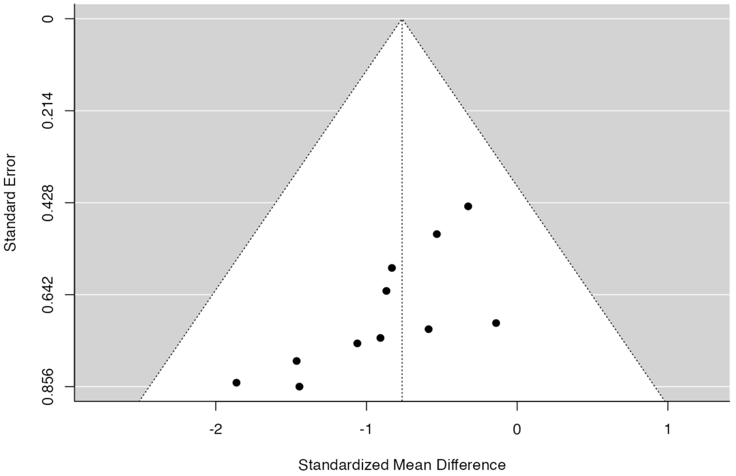

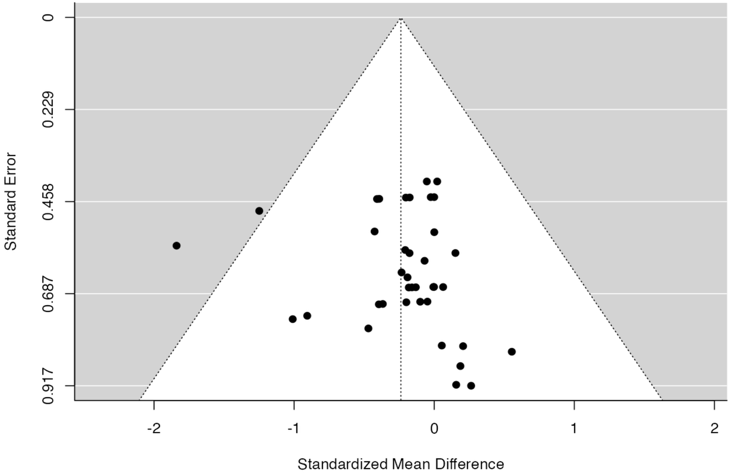
A BM NSG B Spleen NSG


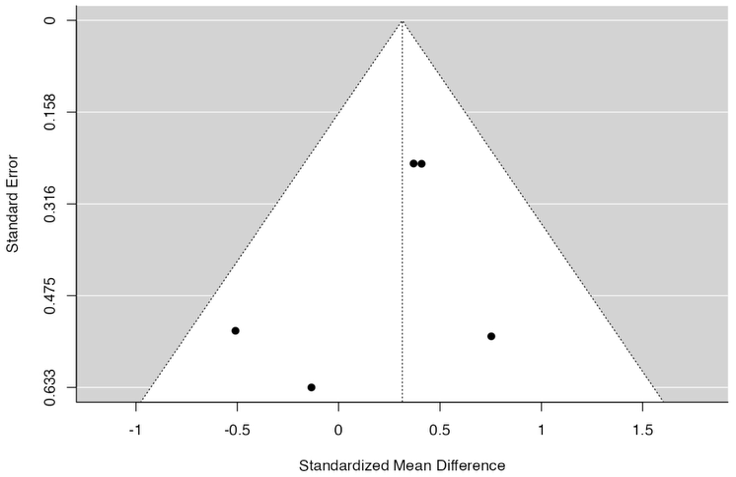
C PB NSGS

**Suppl 8.** Funnel plot of mice strain subgroup analysis. (A) BM engraftment using NSG mice strain reveals neither the rank correlation nor the regression test indicated any funnel plot asymmetry (p = 0.8222 and p = 0.6319, respectively). (B) for spleen engraftment using NSG mice the rank correlation test indicated funnel plot asymmetry (p = 0.0008) but not the regression test (p = 0.0861). (C) for PB using NSGS mice strain neither the rank correlation nor the regression test indicated any funnel plot asymmetry (p = 1.0000 and p = 0.3456, respectively).
