# Supplementary material for: Engraftment Outcome of CRISPR/Cas9-Edited Hematopoietic Stem Cells for Genetic Diseases: A Systematic Review and Meta-Analysis of Preclinical Evidence
Source: J Hematol. 2026 Apr 6;15(2):108–28. doi: 10.14740/jh2190 (PMC13071946; doi:10.14740/jh2190)
Supplement: Suppl 9 — Funnel plot for subgroup route of delivery analysis. [file jh-15-02-108-s009.docx]

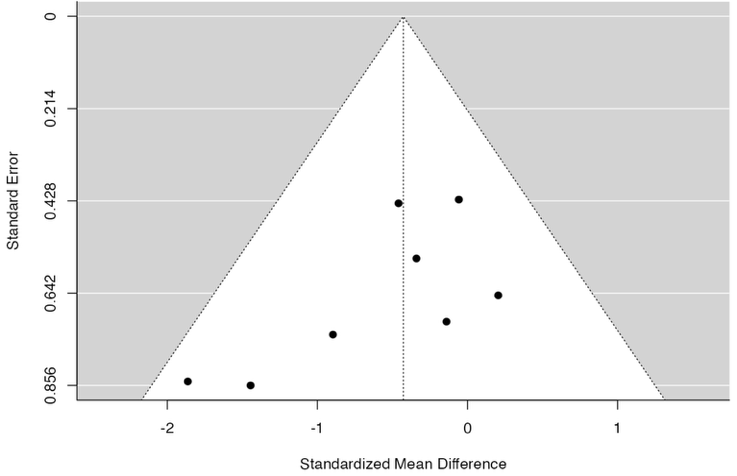

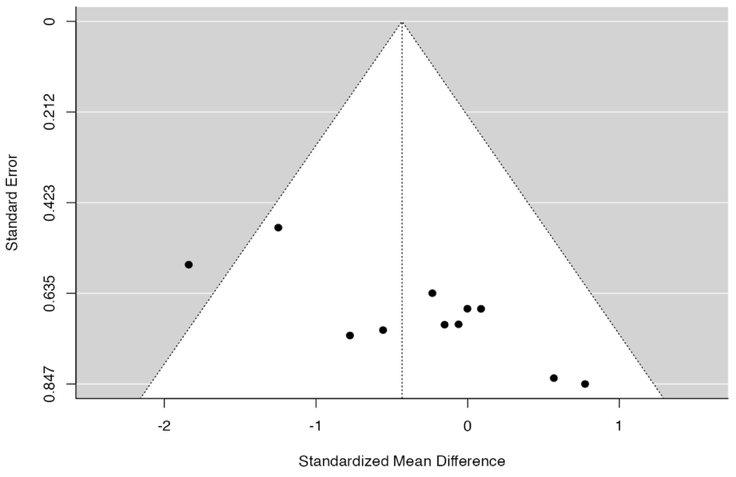
A BM Retro B Spleen Tail vein


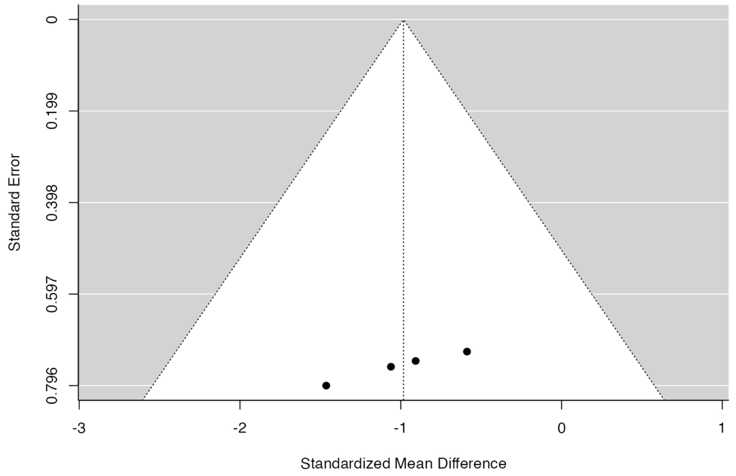

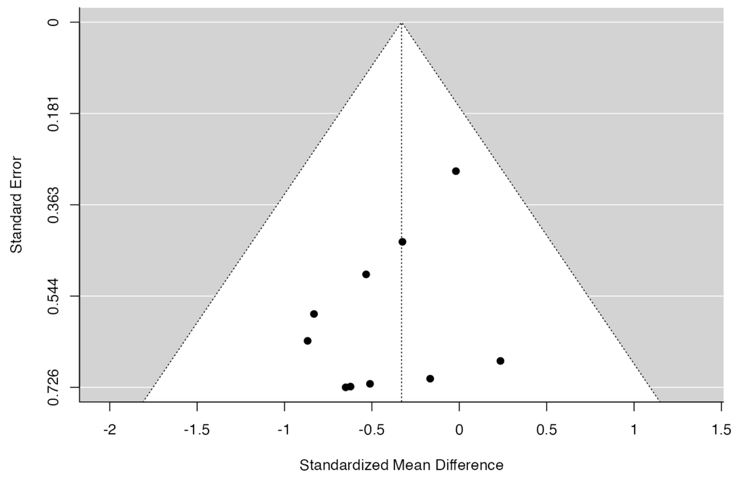
C Spleen Retro-orbital C Spleen Intraperitoneal

**
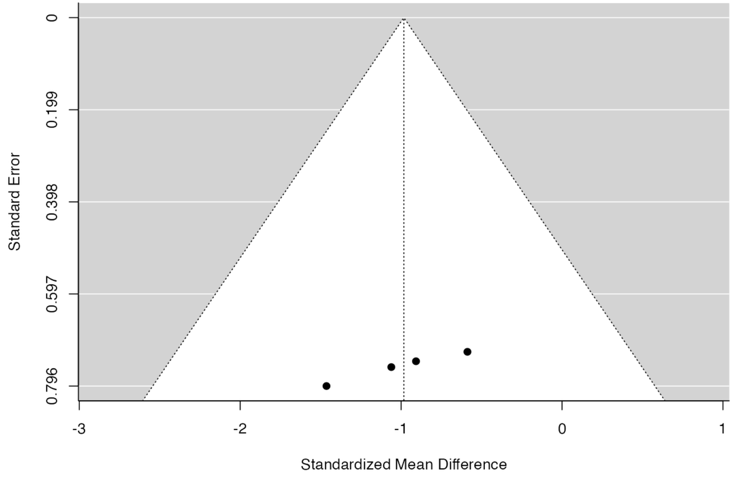
**
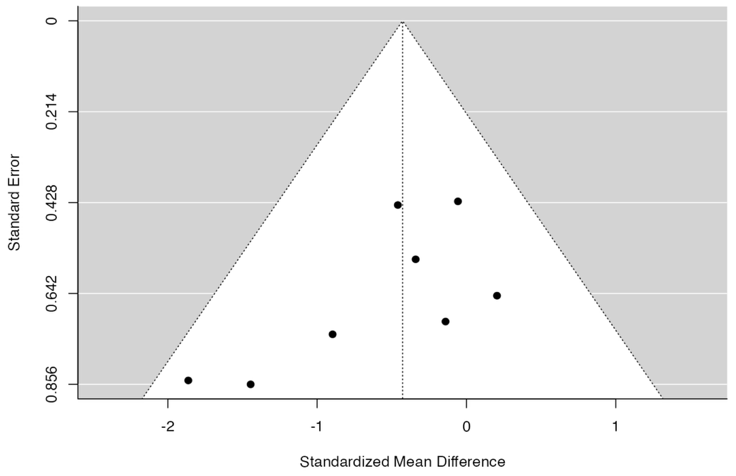
E PB Tail vein F PB Intraperitoneal

**Suppl 9**: Funnel plot for subgroup route of delivery analysis (A) for BM engraftment through retro-orbital route the regression test indicated funnel plot asymmetry (p = 0.0027) but not the rank correlation test (p = 0.1210). Data provided for spleen engraftment through different route of delivery are unbiased as suggested by correlation and regression coefficient respectively for (B) tail vein; p=0.109, p=0.109 (C) retro-orbital; p=0.381. p=0.280 (D) intraperitoneal; p=0.083, p=0.412. The correlation and regression coefficient of data provided for PB engraftment through (E) tail vein; p=0.013, p=0.247 (F) intraperitoneal; p=0.083, p=0.722.
