# Supplementary material for: Engraftment Outcome of CRISPR/Cas9-Edited Hematopoietic Stem Cells for Genetic Diseases: A Systematic Review and Meta-Analysis of Preclinical Evidence
Source: J Hematol. 2026 Apr 6;15(2):108–28. doi: 10.14740/jh2190 (PMC13071946; doi:10.14740/jh2190)
Supplement: Suppl 10 — Funnel plot for the subgroup analysis of peripheral blood as source of HSPC analysis. [file jh-15-02-108-s010.docx]

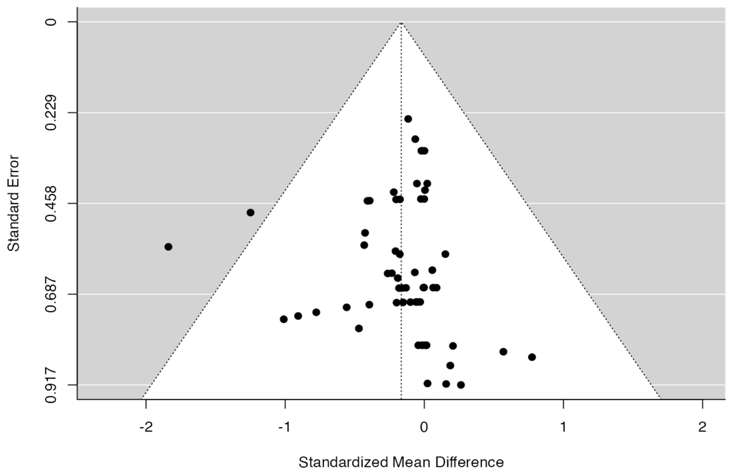

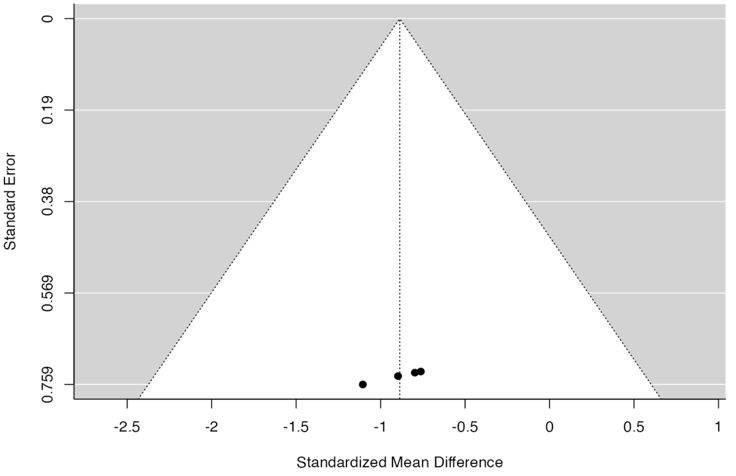
A BM PB B PB PB

**Suppl 10**: Funnel plot for the subgroup peripheral blood as source of HSPC analysis (A) Data provided for bone marrow analysis reveals neither the rank correlation nor the regression test indicated any funnel plot asymmetry (p = 0.9468 and p = 0.8425, respectively). (B) Data provided for PB analysis suggests Neither the rank correlation nor the regression test indicated any funnel plot asymmetry (p = 0.1751 and p = 0.9171, respectively).
