# Supplementary material for: Engraftment Outcome of CRISPR/Cas9-Edited Hematopoietic Stem Cells for Genetic Diseases: A Systematic Review and Meta-Analysis of Preclinical Evidence
Source: J Hematol. 2026 Apr 6;15(2):108–28. doi: 10.14740/jh2190 (PMC13071946; doi:10.14740/jh2190)
Supplement: Suppl 11 — Funnel plot for subgroup analysis of disease-targeted and HDR analyses. [file jh-15-02-108-s011.docx]

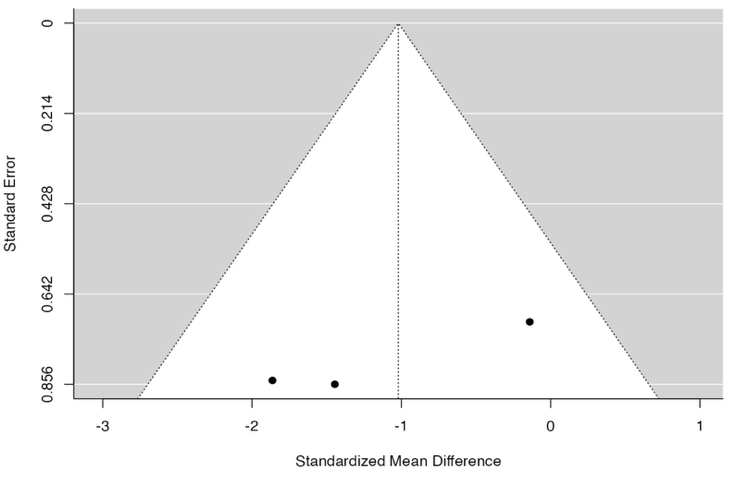

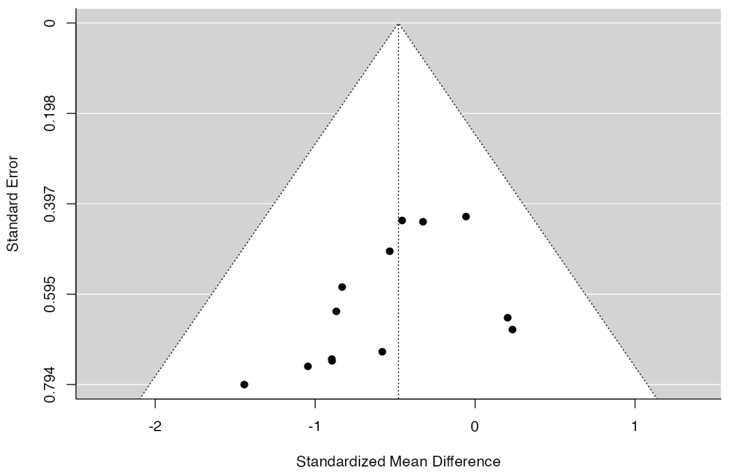
 A Spleen HGP B Spleen Leuk


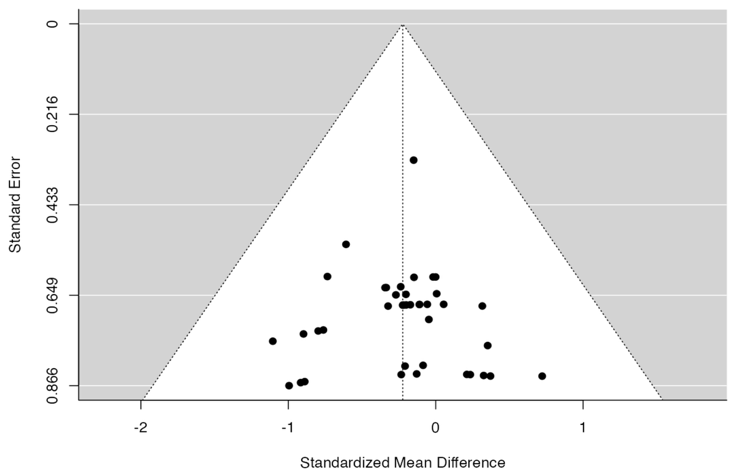
 C PB HGP

**Suppl 11**: Funnel plot for subgroup disease targeted and HDR analysis. The correlation and regression coefficient of data provided for spleen engraftment (A) p=0.030, p=0.199 for HGP (B) p=1.000, p=0.107 for leukaemia. Data provided for PB engraftment (C) targeting HGP disease reveals neither the rank correlation nor the regression test indicated any funnel plot asymmetry (p =0.9539 and p = 0.9776, respectively).
